# Uncovering MicroRNA and Transcription Factor Mediated Regulatory Networks in Glioblastoma

**DOI:** 10.1371/journal.pcbi.1002488

**Published:** 2012-07-19

**Authors:** Jingchun Sun, Xue Gong, Benjamin Purow, Zhongming Zhao

**Affiliations:** 1 Department of Biomedical Informatics, Vanderbilt University School of Medicine, Nashville, Tennessee, United States of America; 2 Division of Neuro-Oncology, Neurology Department, University of Virginia Health System, Charlottesville, Virginia, United States of America; 3 Department of Psychiatry, Vanderbilt University School of Medicine, Nashville, Tennessee, United States of America; 4 Department of Cancer Biology, Vanderbilt University School of Medicine, Nashville, Tennessee, United States of America; University of Edinburgh, United Kingdom

## Abstract

Glioblastoma multiforme (GBM) is the most common and lethal brain tumor in humans. Recent studies revealed that patterns of microRNA (miRNA) expression in GBM tissue samples are different from those in normal brain tissues, suggesting that a number of miRNAs play critical roles in the pathogenesis of GBM. However, little is yet known about which miRNAs play central roles in the pathology of GBM and their regulatory mechanisms of action. To address this issue, in this study, we systematically explored the main regulation format (feed-forward loops, FFLs) consisting of miRNAs, transcription factors (TFs) and their impacting GBM-related genes, and developed a computational approach to construct a miRNA-TF regulatory network. First, we compiled GBM-related miRNAs, GBM-related genes, and known human TFs. We then identified 1,128 3-node FFLs and 805 4-node FFLs with statistical significance. By merging these FFLs together, we constructed a comprehensive GBM-specific miRNA-TF mediated regulatory network. Then, from the network, we extracted a composite GBM-specific regulatory network. To illustrate the GBM-specific regulatory network is promising for identification of critical miRNA components, we specifically examined a Notch signaling pathway subnetwork. Our follow up topological and functional analyses of the subnetwork revealed that six miRNAs (miR-124, miR-137, miR-219-5p, miR-34a, miR-9, and miR-92b) might play important roles in GBM, including some results that are supported by previous studies. In this study, we have developed a computational framework to construct a miRNA-TF regulatory network and generated the first miRNA-TF regulatory network for GBM, providing a valuable resource for further understanding the complex regulatory mechanisms in GBM. The observation of critical miRNAs in the Notch signaling pathway, with partial verification from previous studies, demonstrates that our network-based approach is promising for the identification of new and important miRNAs in GBM and, potentially, other cancers.

## Author Summary

Several recent studies have implicated the critical role of microRNAs (miRNAs) in the pathogenesis of glioblastoma (GBM), the most common and lethal brain tumor in humans, suggesting that miRNAs may be clinically useful as biomarkers for brain tumors and other cancers. However, to date, the regulatory mechanisms of miRNAs in GBM are unclear. In this study, we have systematically constructed miRNA and transcription factor (TF) mediated regulatory networks specific to GBM. To demonstrate that the GBM-specific regulatory network contains functional modules that may composite of critical miRNA components, we extracted a subnetwork including GBM-related genes involved in the Notch signaling pathway. Through network topological and functional analyses of the Notch signaling pathway subnetwork, several critical miRNAs have been identified, some of which have been reinforced by previous studies. This study not only provides novel miRNAs for further experimental design but also develops a novel computational framework to construct a miRNA-TF combinatory regulatory network for a specific disease.

## Introduction

Glioblastoma multiforme (GBM) is the most common and lethal primary brain tumor in humans and is classified as a grade IV astrocytoma by the World Health Organization (WHO) [1]. The tumor is characterized by rapid growth, a high degree of invasiveness, and strong resistance to radiation and chemotherapy [2]. To illuminate its complex characteristics, an understanding of the underlying genetics is critical. During the last decade, numerous genetic studies, including microRNA (miRNA) and mRNA expression profiling, somatic mutation, copy number variation and methylation studies performed by the Cancer Genome Atlas (TCGA) project, and genome-wide association studies (GWAS) by other groups, have substantially contributed to the comprehensive profiling of GBM [3]–[6]. In addition to confirming previous findings, such as *TP53* mutation, *NF1* deletion or mutation, and *EGFR* amplification, these results included several new genetic discoveries such as frequent mutations of the *IDH1* and *IDH2* genes in secondary GBM [3]. Most importantly, these studies support the idea that many of the current risk factors are likely coordinated at the biological pathway or network level rather than at an individual molecular level [6]. Several studies have interrogated networks in the context of gene expression profiles and/or protein interactions to identify novel critical genes and core pathways for GBM, which provides us with new insights into the mechanisms of the disease pathology [7]–[10]. Another important type of biological network, a miRNA-transcription factor (TF) regulatory network, acts as a functional unit in the regulation of cell fate in many cell types and systems, including cancer [11], [12], but this type of network has not yet been systematically investigated in GBM.

In recent years, an increasing number of miRNAs have been identified and linked to cancer [13], [14]. miRNAs are small (∼22 nucleotides) non-coding RNAs that mainly regulate gene expression at the post-transcriptional level in animals [14]. They are involved in cellular development, differentiation, proliferation, apoptosis and tumorigenesis [15], [16]. Similar to other types of cancer, patterns of differential miRNA expression versus normal tissues have been identified for GBM [17]–[19]. For example, several studies consistently confirmed the overexpression of miR-21 in GBM [20]–[24], and several miRNAs are weakly expressed compared with the normal brain, including miR-124, miR-7, and miR-128 [18], [24].

In addition to traditional low-throughput studies, the TCGA project assessed the expression of 534 miRNAs in 240 tumor tissue samples and 10 normal tissue samples. The results have been used to establish GBM subclasses [25], identify miRNA expression signatures to predict GBM patient survival [17], and identify important miRNAs in GBM [26]. These and other studies have made it clear that miRNAs play important roles in GBM, and it appears increasingly likely that miRNAs will be clinically useful as biomarkers and/or therapeutic targets for brain tumors and other cancers [19]. Despite a number of miRNAs reported to be dysregulated in GBM, little is known about which miRNAs play critical roles in the pathology of GBM and their relevant targets [27]. To address these questions, we hypothesized that an investigation of miRNAs in the context of the regulatory transcriptional and post-transcriptional networks will provide a far more comprehensive view of their functional roles in GBM.

TFs regulate gene expression by translating *cis*-regulatory codes into specific gene-regulatory events [28]. Since TFs and miRNAs are both categorized as gene-regulatory molecules and share a common regulatory logic [29], they are capable of cooperatively regulating the same gene: TFs regulate a gene's transcription in the gene's promoter region, while miRNAs regulate a gene's post-transcription in the gene's 3′ untranslated region (UTR). At the network level, it has been demonstrated that the regulation of transcription by TFs and post-transcriptional regulation by miRNAs are tightly coupled [30], [31]. Moreover, the examination of regulatory networks showed that TFs, miRNAs and genes form a combination of transcriptional/post-transcriptional feed-forward loops (FFLs), which comprise over-represented motifs in the mammalian regulatory network [30], [31]. Therefore, the analysis of mixed FFLs in a cellular system has emerged as a powerful tool to understand specific biological events, such as the control of cell fate in many cell types and systems [11].

In a regulatory network, a typical mixed FFL motif contains three components: TF, miRNA and gene. This mixed FFL motif is defined as a 3-node FFL. Considering co-expressed genes may have similar regulation patterns [32], [33], i.e., genes regulated by the same TF and the same miRNA, we hypothesized that inclusion of co-expressed genes in FFL analysis would have more power to detect disease-specific regulatory modules. Accordingly, we extended the 3-node FFL model to a 4-node FFL model, which might complement to the former.

Here, we pursued a regulatory network-based approach for a comprehensive investigation of gene regulation patterns in GBM. This method can be used to identify network modules containing known GBM-related miRNAs and genes. It can also be used to reveal new components for core pathways. Among GBM candidate genes, we identified the potential targets of TFs and GBM-related miRNAs. These datasets and their regulations were used to construct a comprehensive GBM-specific miRNA-TF mediated regulatory network. Furthermore, we constructed the subnetwork from one well-known core pathway in GBM, the Notch signaling pathway, and identified miRNA components involved in it. Based on the network topological analysis and functional analysis, we identified six functionally critical miRNAs in this pathway. Among them, four have been implicated in GBM by previous work. These results demonstrated that the comprehensive GBM-specific miRNA-TF mediated regulatory network contains valuable information for GBM investigators to identify critical miRNAs and their targets for further experimental design, providing further understanding of the regulatory mechanisms of GBM.

## Results

### A novel computational framework for regulatory network construction

One major purpose of this study was to develop an integrative framework for the construction of a comprehensive regulatory network for GBM. This network consisted of feed-forward regulation among three components: GBM-related genes, GBM-related miRNAs and known human TFs. GBM-related genes and miRNAs with evidence of involvement in the pathology of GBM were collected and curated from public databases and literature. For GBM-related genes, we restricted our analyses to the 415 genes with mutation evidence in previous studies (Table S1 and Text S1). For GBM-related miRNAs, we collected 124 mature miRNAs that were reported to be dysregulated in studies assessing miRNA expression only in GBM tissue samples or cell lines. Human TFs were extracted from TRANSFAC Professional (release 2011.4) [34], a manually curated database of eukaryotic TFs, their genomic binding sites and DNA binding profiles. There are five types of regulatory relationships: TF regulation of gene expression (TF-gene) or miRNA expression (TF-miRNA), miRNA repression of gene expression (miRNA-gene) or TF expression (miRNA-TF), and gene-gene coexpression (gene-gene). Each of these regulatory relationships was predicted using computational approaches (Table 1). Considering the disadvantage of these reverse engineering methods, we applied stringent parameters in prediction to obtain high confidence regulations.

**Table 1:** Summary of relationships among GBM-related genes, GBM-related miRNAs, and TFs.

Relationship	Number of pairs	Number of miRNAs^a^	Number of genes	Number of TFs^b^	Method
miRNA-gene^c^	1476	105	214	-	TargetScan
miRNA-TF^d^	2079	103	-	283	TargetScan and TRANSFAC
TF-.gene^e^	6642	-	296	207	Match™
TF-miRNA^f^	1543	65	-	184	Match™
Gene-gene^g^	383	-	112	-	ARACNE

a miRNA: microRNA.

b TF: transcription factor.

c miRNA-gene: miRNA repression of gene expression.

d miRNA-TF: miRNA repression of TF expression.

e TF-gene: TF regulation of gene expression.

f TF-miRNA: TF regulation of to miRNA expression.

g Gene-gene: gene-gene coexpression.

**Figure 1:**
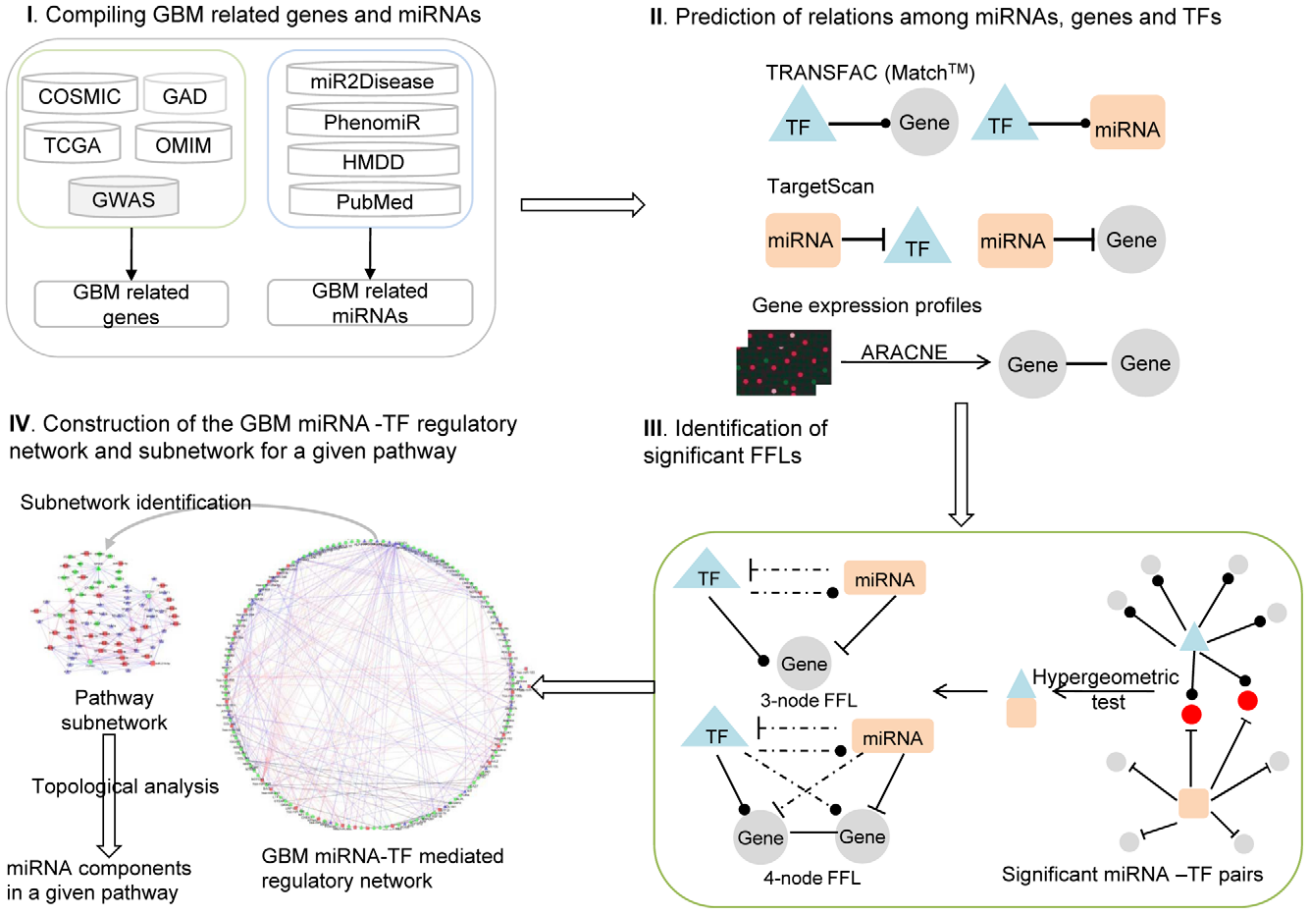
Computational framework for constructing the comprehensive GBM-specific miRNA-TF regulatory network and its application for identifying critical miRNA components in a given pathway. This framework involves four main steps. 1) Data collection. We compiled glioblastoma (GBM)-related genes, GBM-related microRNAs (miRNAs) and known human transcription factors (TFs) from public databases and literature. 2) Regulation prediction. We predicted five types of regulation (TF-gene, TF-miRNA, miRNA-gene, miRNA-TF, and gene-gene coexpression) by integrating TF binding profiles, miRNA target profiles, and gene expression profiles. 3) Identification of significant feed-forward loops (FFLs). Based on the regulation data in step 2, we assembled two types of feed-forward loops (FFLs): 3-node FFLs and 4-node FFLs. 4) Construction of a GBM-specific miRNA-TF regulatory network and performing further subnetwork analyses. By merging the FFLs identified in step 3, we constructed a GBM-specific miRNA-TF regulatory network, which consists of three types of nodes and five types of edges. Furthermore, we extracted subnetworks for core pathways reported for GBM from the GBM-specific regulatory network and predicted the miRNA components involved in these pathways.

To integrate these regulations into a miRNA-TF regulatory network, we only included FFLs with significant miRNA-TF pairs, pinpointed by the hypergeometric test, that potentially cooperate in regulating the same targets. Based on the combinatory regulatory network, we performed further analyses of the network topological properties and functional associations to identify critical miRNAs (see Figure 1 for the framework and the Materials and Methods for details). It is necessary to point out that, in this computational framework, a novel FFL model (4-node model) was developed for the construction of the regulatory network. To illustrate that the framework has a promising application in cancer investigation, in this study, we focused on the GBM regulatory network and identified the miRNA components for the Notch signaling pathway. The analyses illustrated the framework is promising for further identification of critical miRNAs in the pathology of cancer.

### Highly confident regulatory relationships among miRNAs, genes and TFs

Table 1 summarizes the five types of potential regulatory relationships mentioned above and their related methods. We provide more details below.

#### miRNA-gene and miRNA-TF repression

We predicted miRNA targets in genes by parsing TargetScan prediction results [35] and filtered out the false positive assignments of miRNAs to genes by applying stringent requirements (see Materials and Methods). Consequently, among 415 GBM-related genes, 214 were potential targets of 105 miRNAs of our compiled 124 GBM-related miRNAs; they formed 1,476 miRNA-gene pairs. Among the 214 target genes, the top genes targeted by the largest number of GBM-related miRNAs were *DLGAP2* and *SOX11*, which were targeted by 33 GBM-related miRNAs. Among the 105 GBM-related miRNAs, the miRNA that targeted the largest number of GBM-related genes was miR-340. To test whether we observed more GBM miRNA targets in the 415 GBM-related genes than the randomly selected 415 genes, we performed a permutation to count the number of targets of each GBM miRNA in the same number of genes (415 genes), which were randomly selected from human protein-coding genes. We repeated this process 10,000 times to obtain an empirical *P*-value. Most of the miRNAs had a significantly larger number of targets in these genes than randomly selected genes (t-test, *P*-value = 1.38×10^−5^). Using the same miRNA target prediction method, we screened the miRNA targets of 428 human TFs. We obtained 2,079 miRNA-TF pairs among 103 GBM-related miRNAs and 283 TFs. Among the 103 GBM-related miRNAs, the miRNA that targeted the largest number of TFs (i.e., 60 TFs) was miR-124. Among the 283 TFs, the TF gene targeted by the largest number of miRNAs (i.e., 44 miRNAs) was *NFAT5*.

#### TF-gene and TF-miRNA regulation

To find the regulation of TF to genes or miRNAs, we explored the TFs and their binding profiles from the TRANSFAC Professional database and predicted TF binding sites using its Match™ software [36] by applying stringent criteria (see Materials and Methods). For a TF, if there is one binding site within the transcription start site (TSS) proximal region of a gene (from 1 kb upstream to 500 bp downstream), the gene was defined as the target of the TF. We thus identified 296 GBM-related genes as targets of 207 TFs, which formed a total of 6,642 TF-gene pairs. In these pairs, the range of number of genes regulated by one TF was from 1 to 245. Among the 207 TFs, the TF that targeted the largest number of GBM-related genes was *PAX4* (paired box 4). *PAX4* plays critical roles during both fetal development and cancer growth [37].

As with the GBM-related genes, we applied the same prediction process to identify miRNA targets of TFs, since previous studies have shown that miRNA expression is regulated in a similar manner to protein-coding genes [38]. We identified 1,543 TF-miRNA pairs, which consisted of 65 GBM-related miRNAs and 184 human TFs. Among these pairs, the range of the number of TFs potentially targeting one miRNA was from 7 to 65. miR-9 was predicted to be targeted by 65 TFs. The range of the number of miRNAs targeted by a TF was from 1 to 58. The TF that targeted the largest number of GBM-related miRNAs was ELF1 (E74-like factor 1).

#### Gene-gene coexpression

Based on the filtered gene expression profiles from three different microarray platforms [39], we identified 383 co-regulated genes using the software ARACNE [40] (see Materials and Methods). The degree of coexpression ranged from 1 to 23. Specifically, gene *CAST* co-expressed with 23 other genes; this particular gene is involved in numerous membrane fusion events, such as neural vesicle exocytosis and platelet and red-cell aggregation [41].

### Significant 3-node and 4-node feed-forward loops

**Figure 2:**
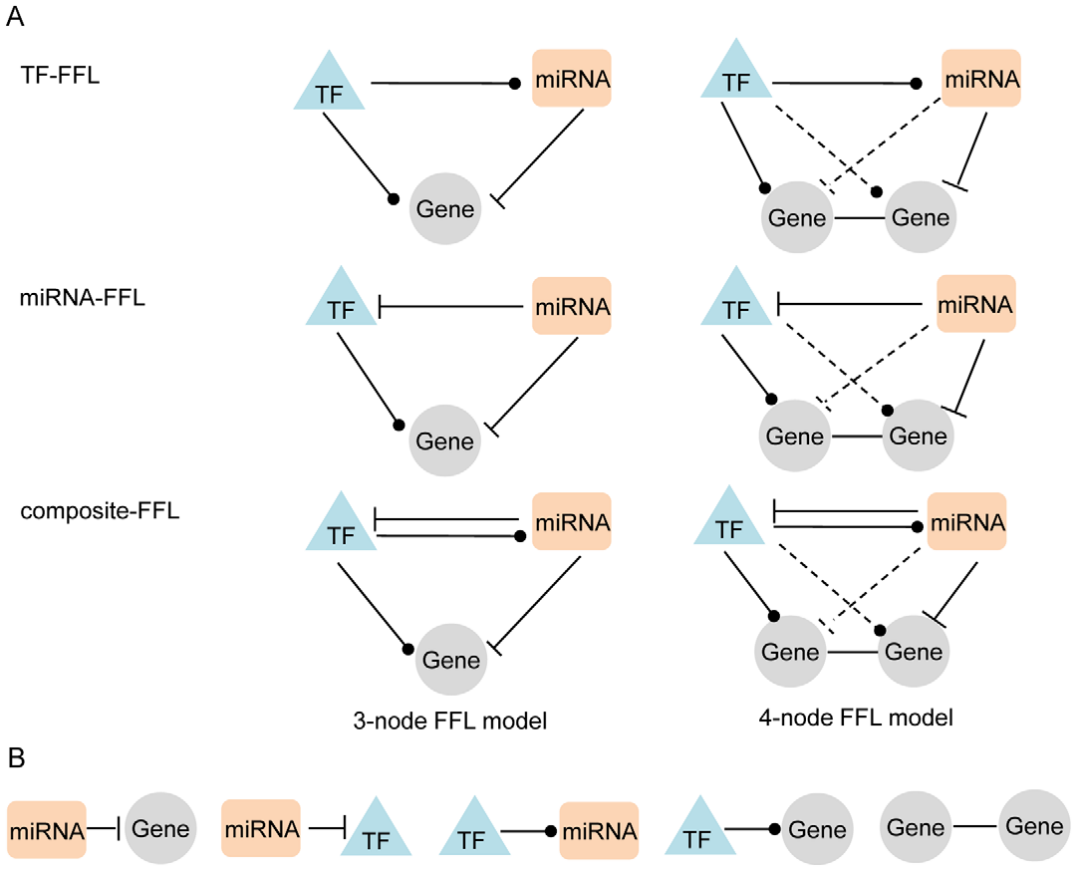
A catalogue of mixed feed-forward regulatory loops (FFLs). According to the relationship between the transcription factor (TF) and microRNA (miRNA), the mixed FFLs were classified as the TF-FFL model (the TF directly regulates the miRNA), miRNA-FFL model (the miRNA only directly regulates the TF) or composite-FFL model (the TF and the miRNA regulate each other). The relationships represented by solid lines are required while the relationships represented by dot lines are not required. B) Five types of putative regulations involved in these FFLs: miRNA-gene represents that the miRNA represses gene expression; miRNA-TF represents that the miRNA represses the TF gene expression; TF-gene represents the regulation by TF of the expression of the gene; TF-miRNA represents the regulation of TF to expression of miRNAs; and, gene-gene represents gene coexpression.

FFLs have been demonstrated as one of the most common types of transcriptional network motifs [42]. Typically, a FFL consists of three components: a miRNA, a TF, and a joint target, which is defined as a 3-node FFL. In this study, we expanded the 3-node FFL model to a 4-node FFL model to explore more regulatory modules. Figure 2A shows the detailed relationships in these FFLs. According to the regulatory relationship between two regulators (TF and miRNA) in each FFL, we classified FFLs into 3 types: TF-FFL, miRNA-FFL and composite FFL (Figure 2). Specific to the 3-node FFLs, the TF-FFL model includes TF regulation of a miRNA and a gene, and it also includes miRNA repression of a target gene. The miRNA-FFL model includes miRNA repression of both a target gene and a targeted TF, as well as TF regulation of a target gene. The composite-FFL model includes TF regulation of both a miRNA and a target gene, as well as miRNA repression of the TF gene and the target gene. The three types of FFLs are exclusive to each other. For 4-node FFLs, the design is similar to the 3-node FFL model, but each TF or miRNA may regulate both co-expressed genes.

Furthermore, we merged those FFLs with the same TF-miRNA regulation. Thus, the merged FFLs composed of a known TF, a mature miRNA, and a list of GBM-related genes or a list of GBM co-regulated gene pairs (Figure S1). Table 2 summarizes the number of nodes and links in the 3-node and 4-node FFLs.

**Table 2:** Summary of 3-node and 4-node feed-forward loops based on glioblastoma related data.

		Number of nodes^b^			Number of links^b^					
Motif	Number of merged FFLs^a^	Genes	miRNAs	TFs	Total	TF-gene	Gene-gene	miRNA-gene	miRNA-TF	TF-miRNA
***3-node***										
TF-FFL	656	144	59	122	2473	1227	-	590	0	656
miRNA-FFL	432	131	84	83	1768	592	-	744	432	0
Composite-FFL	40	76	24	23	344	130	-	134	40	40
Total	1128	153	97	135	3709	1570	-	971	472	696
***4-node***										
TF-FFL	482	51	46	90	1144	438	99	125	0	482
miRNA-FFL	299	53	78	62	775	189	88	199	299	0
Composite-FFL	24	40	17	14	173	47	43	35	24	24
Total	805	55	80	105	1647	494	104	220	323	506

a FFL: feed-forward loop.

b Definitions of the nodes and links were provided in Table 1.

#### 3-node FFLs

Starting from two types of regulation, miRNA-gene and TF-gene, we assembled all possible miRNA-gene-TF units (relationships). After filtering the random TF-miRNA pairs using the hypergeometric test based on the common targets of miRNAs and TFs [43], we obtained a total of 3,914 unique FFLs, which grouped to 1,128 merged FFLs based on the definition in Figure S1 (Table S2). The merged FFLs involved a total of 153 GBM-related genes, 97 GBM-related miRNAs and 135 TFs. The number of targets in these merged FFLs ranged from 1–15, and 70.74% of the 3-node FFLs targeted up to 2 genes.

For the genes targeted by the TF and miRNA from a merged FFL (co-targeted genes), we examined if they have more similar function than randomly selected genes from all human genes. We computed Gene Ontology (GO) semantic similarity scores using the R GOSemSim package [44]. For GO categories biological process (BP), molecular function (MF), and cellular component (CC), the semantic scores of these genes in 3-node FFLs tended to significantly skew towards higher scores than those of randomly selected genes (Figure S2). We further tested whether the targets in each merged FFL tended to belong to the same protein family than randomly selected human proteins using the Pfam annotations [45] (Text S2). We found that the numbers of target pairs from 3-node FFLs with the same Pfam annotation were significantly higher than those from the randomly selected genes (Table S3). The results indicated that the genes regulated by the same TF-miRNA pairs in 3-node FFLs were more likely to share Pfam annotation than randomly selected genes (Fisher's exact test, *P*-value<2.2×10^−16^). In summary, according to these function and protein family similarity analyses, the co-targeted genes tended to participate in the same biological processes, locate in the same cellular components, or belong to the same protein family.

Among the 1,128 merged 3-node FFLs, 656 (58.16%) belonged to TF-FFLs, 432 (38.30%) belonged to miRNA-FFLs, and 40 (3.54%) belonged to composite-FFLs. The numbers of nodes and links in composite-FFLs were much smaller than those in TF-FFLs and miRNA-FFLs. However, the 40 composite-FFLs (3.54%) were comprised of 24 (24.74%) GBM-related miRNAs and 23 (17.04%) TFs regulating 76 (49.67%) GBM-related genes (Table 2), indicating that only a few composite-FFLs recruited nearly half of GBM-related genes via a few regulators. Additionally, among the 76 genes regulated by the composite-FFL model, 72 (94.74%) of GBM-related genes in composite-FFLs were also regulated through the TF-FFL and miRNA-FFL models (Figure S3). Similarly, most of the TFs and miRNAs (87.50% and 95.65%, respectively) involved in composite-FFLs participated in the other two regulation models. These observations suggest that most of the GBM-related genes could be regulated in multiple ways. To further illustrate the functional importance of the 72 genes, we performed pathway enrichment analyses using the software WebGestalt [46]. Thirteen KEGG pathways were significantly enriched. Interestingly, 9 were within the top 10 enriched KEGG pathways identified using the 153 genes in all 3-node FFLs (Table S4), further suggesting the efficiency of composite-FFLs.

#### 4-node FFLs

Using a process similar to the 3-node model, we identified a total of 2,042 4-node FFLs, each of which included a human TF, a GBM miRNA and two co-expressed genes in GBM. These FFLs were grouped as 805 merged FFLs, each of which was composed of a known TF, a mature miRNA and a list of GBM co-regulated genes (Table S5). These 805 merged FFLs involved a total of 55 GBM-related genes, 80 miRNAs and 105 TFs. The number of targets in these merged FFLs ranged from 1–13, and 44.10% of the 4-node FFLs targeted up to 2 pairs of co-regulated GBM-related genes. Similarly, these co-targeted gene pairs tended to have higher pair-wise functional similarity scores (Figure S2) or had significantly more gene pairs sharing Pfam annotations (Table S3) than randomly selected gene pairs (Fisher's exact test, *P*-value<2.2×10^−16^). The results again indicated that co-targeted genes in 4-node FFLs tended to have more similar functions than the randomly selected genes. Additionally, compared to co-targeted genes in 3-node FFLs, genes in 4-node FFLs tended to have higher similarity scores or have more gene pairs sharing Pfam annotations. This comparison indicated that genes in 4-node FFLs might have stronger functional relationship than those in 3-node FFLs.

Among the 805 merged FFLs, 482 (59.88%) belonged to TF-FFLs, 299 (37.14%) belonged to miRNA-FFLs and 24 (2.98%) belonged to composite-FFLs. Similar to the 3-node FFLs, the 24 (2.98%) 4-node composite-FFLs were composed of 17 (20.00%) GBM-related miRNAs and 14 (10.07%) TFs, which regulated 40 (72.73%) GBM-related genes. Forty (100%) of the GBM-related genes were also regulated through the TF-FFL and miRNA-FFL models (Figure S3). Additionally, most of the regulatory elements (TFs: 100% and miRNAs: 85.71%, respectively) also participated in the other two regulation models. This result further supported that a few composite-FFLs could recruit the majority of GBM-related genes via a few regulators, as observed in the 3-node FFL analysis above.

#### 4-node FFLs complement 3-node FFLs

To further explore the relationship between 4-node FFLs and 3-node FFLs, we examined their nodes and edges. We observed that the number of nodes in 4-node FFLs was less than that in 3-node FFLs (Table 2). Figure S4A summarized the overlap of the three types of nodes between 3-node FFLs and 4-node FFLs. For GBM-related genes, 32 were shared by both types of FFLs. These genes accounted for only 18.18% of the total 176 genes. We performed a pathway enrichment analysis to examine if these 32 genes have any biological bias with the reference of whole human protein-coding genes. Ten KEGG pathways were found to be significantly enriched with these genes (adjusted *P*-value<0.05). All of these pathways were related to cancer: “Pathways in cancer,” “Focal adhesion,” “ECM-receptor interaction,” “Colorectal cancer,” “Cytokin-cytokin receptor interaction,” “Endocytosis,” “Renal cell carcinoma,” “Pancreatic cancer,” “Glioma,” and “Melanoma.” Among them, 7 could be found in the top 10 pathways enriched in the 176 genes (Table S6). The 4-node FFLs recruited an additional 23 GBM-related genes. Pathway analysis found these genes were enriched in two pathways (“ECM-receptor interaction” and “Focal adhesion”) detected by the common 32 genes, as well as three other pathways (“Leukocyte transendothelial migration,” “Lysosome,” and “Regulation of actin cytoskeleton”). These comparisons indicated that, compared to 3-node FFLs, the 4-node FFLs could recruit genes that are not only directly involved in cancer related pathways but also associated with cell motility and cell proliferation, both of which have been implicated in the pathology of glioma [47]. In contrast, the majority of miRNAs (78 out of 99 miRNAs, 78.79%) and TFs (98 out of 142 TFs, 69.01%) were recruited by both the 4-node and 3-node FFLs, indicating that miRNAs and TFs had no significant difference between these two types of FFLs.

Figure S4B summarizes the overlap of the four types of links (TF-gene, miRNA-gene, miRNA-TF, and TF-miRNA) between 3-node FFLs and 4-node FFLs. We observed that the majority of TF-gene links (293 out of 494, 59.31%) in 4-node FFL had not been covered by 3-node FFLs, while the majority of miRNA-gene (158 out of 220, 71.82%), miRNA-TF (245 out of 323, 75.85%), and TF-miRNA (398 out of 506, 78.66%) links were covered by 3-node FFLs. Overall, these observations suggested that 4-node FFLs could recruit novel GBM-related genes and novel regulatory relationships, which might complement 3-node FFLs.

### A GBM-specific miRNA-TF mediated regulatory network

After converging the significant 3-node and 4-node FFLs identified in the previous subsection, we constructed a miRNA-TF mediated regulatory network for GBM, the major biological output of our computational analysis. The resultant network contained a total of 4,354 edges and 408 unique nodes (Table S7). Among the 4,354 edges, 1,033 belonged to miRNA-gene pairs, 550 to miRNA-TF pairs, 1,863 to TF-gene pairs, 804 to TF-miRNA pairs, and 104 to gene-gene pairs. Among the 408 nodes, 176 belonged to GBM-related genes, 99 to GBM-related miRNAs and 142 to human TFs. Among GBM-related genes and TFs in this regulatory network, 9 genes overlapped (*ARNT*, *FLI1*, *FOXO3*, *FOXO4*, *GATA3*, *SMAD4*, *STAT3*, *TCF12*, and *ZEB1*). Although the network only recruited 176 (43.46%) of the 415 GBM-related genes and 99 (79.84%) of the 124 GBM-related miRNAs, given the uncertainty of associations between candidate genes and the disease, we regarded it as a representation of the regulatory network in GBM.

To provide a general view of this regulatory network, we calculated degrees (connectivity) and their distribution, which are basic topological network measures [47]. In this complicated network, degree values of genes, miRNAs and TFs ranged from 2 to 66, 2 to 77, and 2 to 123, respectively. The average degrees of genes, miRNAs and TFs were 18.70, 24.11, and 23.80, respectively. The degree distribution for genes, miRNAs and TFs were strongly right-skewed, indicating that most nodes had a low degree, while only a small portion of nodes had a high degree (Figure S5). Therefore, we observed only a few miRNAs, GBM-related genes and TFs exhibited a high degree in the network. In the context of this regulatory network, these molecules act as hubs that might play important roles in GBM.

Hubs are highly connected nodes in a network, suggesting critical roles in maintaining the overall connectivity of the network [47]. Consistently, hubs in the PPI network are more likely to be essential genes [48], [49]. Using the hub definition method proposed by Yu et al. [50], we determined the degree cutoff values 38, 49, and 71 for genes, miRNAs and TF hubs, respectively. Accordingly, we identified 15 hub genes (*FOXO3*, *SMAD4*, *TCF12*, *BCL11A*, *PDGFRA*, *KLF4*, *NRAS*, *SOX11*, *CACNA1E*, *ELAVL2*, *PIK3R1*, *RPS6KA3*, *SLC9A2*, *CYLD*, and *PTCH1*), 4 hub miRNAs (miR-9, let-7i, miR-495 and miR-130a) and 6 hub TFs (TEAD1, SP1, MZF1, NEUROD1, GATA1, and TCF7). Among them, genes *PIK3R1* and *PDGFRA* had been reported to have high mutation frequencies in 91 GBM samples (9% and 13%, respectively), and are involved in the RTK/PI3K signaling pathway, a core GBM pathway [6].

**Figure 3:**
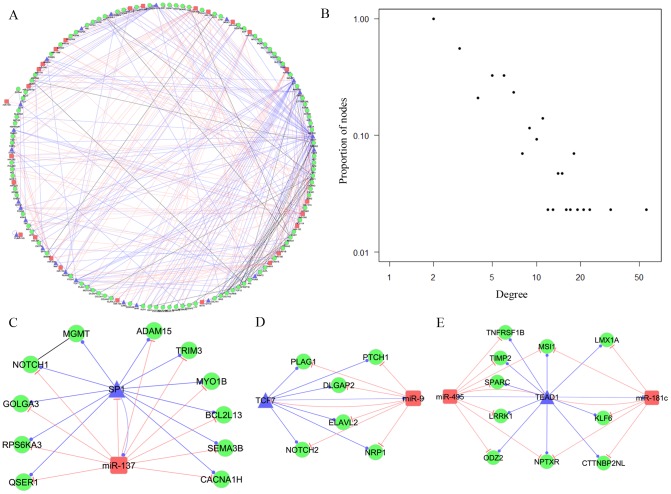
Graphical representations of the composite glioblastoma-related miRNA-TF regulatory network and its network characteristics. A) Graphical representation of the composite glioblastoma miRNA-TF regulatory network. The network was generated from 3-node and 4-node composite-FFL motifs. B) Degree distribution of all nodes (genes, miRNAs and TFs) in the network. The Y-axis represents the proportion of nodes with a specific degree. C–E) Three higher-order subnetworks. In each subfigure, nodes in red correspond to GBM-related miRNAs, nodes in green correspond to GBM-related genes, and nodes in blue correspond to transcription factors. The edge colors represent different relationships: red for the repression of miRNAs to genes or TFs, blue for the regulation of TFs to genes or miRNAs, and black for the coexpression of GBM-related genes.

In the above FFL analyses, we noticed that composite 3-node and 4-node FFLs recruited the most GBM-related genes in each category (49.67% and 72.73%, respectively), which indicated that composite-FFLs could play important roles in regulating GBM candidate genes. Therefore, we converged these composite-FFLs and generated a regulatory subnetwork that only included composite-FFLs. The resulting subnetwork included 457 edges and 101 GBM-related genes, which accounted for 57.38% of GBM-related genes (176) in the GBM-specific miRNA-TF mediated regulatory network and were regulated by only 26 GBM-related miRNAs (24.24%) and 24 TFs (16.90%). We defined this subnetwork as the composite miRNA-TF regulatory network in GBM; it could provide a main framework for the regulatory systems involved in GBM (Figure 3A). In this regulatory network, the distribution of all nodes was again strongly right-skewed; that is, only a few nodes had high degree in the network (Figure 3B). Using the same method to define hubs, we identified four hub genes (*NRP1*, *FOXO3*, *SMAD4*, and *TNFRSF1B*), six hub miRNAs (miR-495, miR-9, miR-137, miR-30d, miR-181c, and miR-30e), and three hub TFs (TEAD1, SP1, and ZBTB7A). Previously, Zhang et al. [51] proposed that a higher-order network structure is a frequently observed motif in integrated mRNA-protein networks. In our regulatory network, we also found several miRNAs and TFs involved in higher-order subnetworks. For instance, we identified three higher-order composite subnetworks. The first one (Figure 3C) included one hub TF (SP1) and one hub miRNA (miR-137), which together regulated 10 genes. The second composite subnetwork included one TF, one hub miRNA, and 6 genes (Figure 3D). The third one included one hub TF, two hub miRNAs, and 12 genes (Figure 3E).

We further examined enriched pathways in these 101 GBM-related genes involved in the GBM composite regulatory network. This further examination was important, as biological pathways that are statistically enriched in a set of disease genes may provide important cellular process information for our understanding of the molecular pathology of the disease. For the 101 genes, we identified 39 pathways that were significantly enriched (adjusted *P*-value<0.01) (Table 3). Among these 39 pathways, 10 (25.6%) were directly related to cancer, including glioma and GBM. Several are well-known core pathways involved in GBM, such as PTEN signaling, PI3K/AKT signaling and Notch signaling.

**Table 3:** Canonical pathways overrepresented in genes involved in the composite glioblastoma-specific regulatory network.

Ingenuity canonical pathways	Adjusted *P*- value^a^
Hepatic fibrosis/hepatic stellate cell activation	3.16×10^−11^
Molecular mechanisms of cancer	1.51×10^−5^
Pancreatic adenocarcinoma signaling	1.74×10^−5^
PTEN signaling	1.74×10^−5^
Melanocyte development and pigmentation signaling	0.0005
CNTF signaling	0.0006
Chronic myeloid leukemia signaling	0.0007
Glioma signaling	0.0007
Glioblastoma multiforme signaling	0.0007
Glucocorticoid receptor signaling	0.0007
Renal cell carcinoma signaling	0.0014
NF-κB signaling	0.0014
PI3K/AKT signaling	0.0015
HER-2 signaling in breast cancer	0.0017
Prostate cancer signaling	0.0019
Insulin receptor signaling	0.0019
Human embryonic stem cell pluripotency	0.0021
Neuregulin signaling	0.0025
IL-6 signaling	0.0032
PPAR signaling	0.0032
IGF-1 signaling	0.0037
Antiproliferative role of TOB in T cell signaling	0.0050
Acute phase response signaling	0.0050
IL-15 signaling	0.0050
ERK5 signaling	0.0050
Role of JAK1 and JAK3 in γc cytokine signaling	0.0054
Axonal guidance signaling	0.0054
Non-small cell lung cancer signaling	0.0054
Growth hormone signaling	0.0059
Macropinocytosis signaling	0.0059
Intrinsic prothrombin activation pathway	0.0059
Neurotrophin/TRK signaling	0.0059
Small cell lung cancer signaling	0.0062
PDGF signaling	0.0062
FLT3 signaling in hematopoietic progenitor cells	0.0063
Prolactin signaling	0.0065
Ceramide signaling	0.0081
Notch signaling	0.0095
TGF-β signaling	0.0098

a Adjusted *P*-value was calculated by Fisher's exact test following by Benjamini-Hochberg multiple testing correction.

### Identification of miRNA components in the Notch signaling pathway in GBM

To demonstrate that the GBM-specific miRNA-TF mediated regulatory network is useful to identify miRNA components for core pathways, we took a convergent strategy to narrow down the candidate list. We first generated subnetworks for core pathways in GBM and then performed network characteristic analyses, including degree and degree distribution, hub, network modularity, to identify key components. Aside from degree of the node and degree distribution and hub definition mentioned before, the most frequently used approach for biological network analysis is to cluster or partition the whole network into subcomponents, i.e., modularity. Previous studies have revealed that highly connected groups of proteins tend to participate in the same biological process or complex [52]. In this study, we selected the Notch signaling pathway as an example to illustrate that the network is a useful resource for hypothesis generation and that our computational framework is promising.

**Figure 4:**
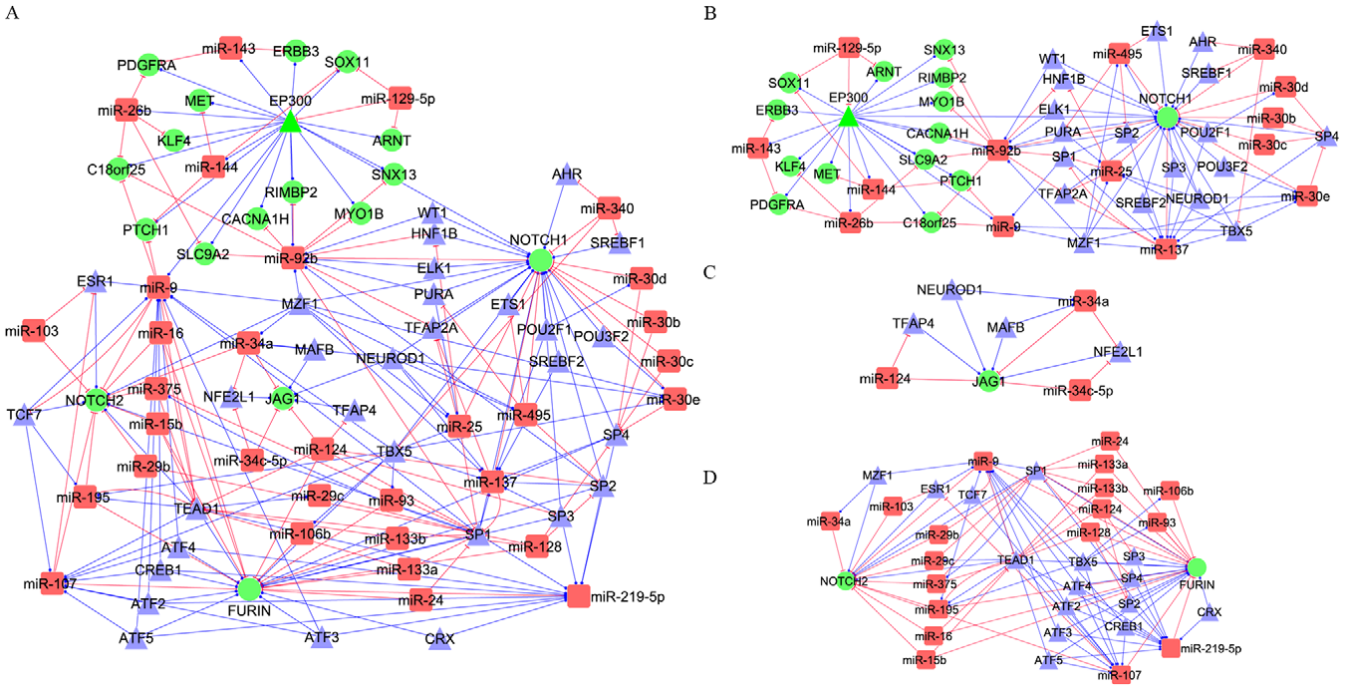
Notch-specific miRNA-TF regulatory network and its subnetworks related to GBM. A) Notch-specific miRNA-TF regulatory network related to GBM. B) GBM gene-centered subnetwork. The subnetwork includes most of the GBM-related genes involved in the Notch-specific miRNA-TF regulatory network. C) Centered subnetwork. The subnetwork links the GBM gene-centered subnetwork and the GBM regulator-centered subnetwork. D) GBM regulator-centered subnetwork. Except for two nodes, 33 nodes are GBM-related miRNAs and human TFs. Definition of colors and shapes for nodes and edges is the same as in Figure 3.

The Notch signaling pathway strongly influences stem cell maintenance, development and cell fate [53]. Growing evidence indicates it plays a key role in cancer, including gliomas [54], [55]. According to pathway information recorded in the KEGG database [56] and Ingenuity Canonical Pathways (http://www.ingenuity.com/), there were five genes in the GBM miRNA-TF mediated regulatory network that belonged to the Notch pathway: *EP300*, *NOTCH1*, *NOTCH2*, *FURIN*, and *JAG1*. We generated a subnetwork for these 5 genes by merging the FFLs that included at least one of these five genes (Figure 4A). We defined it as the GBM Notch-specific miRNA-TF regulatory network, which included 222 edges, 17 GBM-related genes, 32 GBM-related miRNAs and 31 TFs. These 32 miRNAs might be involved in the Notch signaling pathway, providing a potential pool for further experimental determination of miRNAs involved in this pathway (Table S8). We noticed that there was no 4-node FFL involved in the GBM Notch-specific regulatory network.

To identify the critical candidates from the above 32 miRNAs, we further evaluated their importance based on network topological and functional analyses. The degree distribution of all nodes in this subnetwork was also strongly right-skewed. Using the same method to identify the hubs above, we identified four GBM hub genes (*NOTCH1, FURIN*, *NOTCH2*, and *EP300*), four hub miRNAs (miR-9, miR-92b, miR-137 and miR-295-5p) and four hub TFs (EP300, SP1, TEAD1, and TBX5). Thus, the network global property analysis indicated that these four hub miRNAs might play important roles in the Notch signaling pathway.

To investigate other miRNAs in the GBM Notch-related miRNA-TF regulatory network, we used the software CFinder [57] to identify tightly connected subnetworks. CFinder is a popular network analysis tool for examination of nodes' distributions in networks and communities. We obtained four communities in the Notch regulatory network. The first one (Figure S6A) included 15 GBM-related genes, 14 GBM-related miRNAs and 18 TFs. Since the subnetwork included the most GBM-related genes (88.2%) involved in the GBM Notch related regulatory network, we called this subnetwork the gene-centered subnetwork. The second community (Figure S6B) includes two GBM-related genes, 17 GBM-related miRNAs and 15 TFs. Since most of the nodes in this subnetwork are regulators, we defined it as the regulator-centered subnetwork. The third one includes one GBM-related gene, two miRNAs, and three TFs (Figure S6C); the last one includes one GBM-related gene, one miRNA and one TF (Figure S6D). Considering that the last two subnetworks had one common GBM-related gene, *JAG1*, and both were located in the center of the Notch-specific network, we merged these subnetworks together and defined it as a centered subnetwork (Figure 4). Consequently, three Notch-specific subnetworks were identified (Figure 4B, 4C, and 4D).

The centered subnetwork included 8 nodes, none of which belonged to the hubs we identified above. When the centered subnetwork was removed, the connection between the other two subnetworks was lost (Figure S7). To further examine this feature, we removed the nodes directly linked to the centered subnetwork; most parts of the Notch regulatory network were loosely connected except among GBM-related genes (Figure S8). These local network analyses showed that the centered subnetwork could serve as a bridge subnetwork and play an important role in the development of GBM. To further examine the role of the centered subnetwork, we used a GO enrichment analysis to identify biological processes associated with the three subnetworks. The gene-centered subnetwork mainly corresponded to the development processes. The centered subnetwork corresponded to regulation of biological processes and developmental processes. The regulator-centered subnetwork corresponded to regulation of biological processes and metabolic processes. These functional association analyses revealed that the centered subnetwork could play the central role in this subnetwork. Based on the important role of this centered subnetwork in the Notch-specific pathway, and two miRNAs, miR-124 and miR-34a, which have direct connections with two other subnetworks, we proposed that these two miRNAs might play important roles in the Notch signaling pathway involved in GBM.

In summary, based on the network topological analysis of the GBM Notch regulatory network and its subnetworks, we identified 32 human miRNAs that might be involved in the Notch signaling pathway, and six of them (miR-124, miR-137, miR-219-5p, miR-34a, miR-9, and miR-92b) might play important roles in this pathway.

## Discussion

In this study, we explored the combinatory regulation of miRNAs and TFs that have an impact on genes involved in the pathology of GBM. We developed a computational framework to construct and analyze a regulatory network for complex diseases. Our framework started with a compilation of numerous data sources to identify disease candidate genes and miRNAs and then inferred regulatory relationships using a large panel of computational tools. Based on these relationships, we focused on 3-node FFLs and 4-node FFLs to generate a GBM-specific regulatory network. This unique computational framework illustrated that it is indeed possible to process multiple types of data (e.g., mutation data, gene expression data, and knowledgebase) by combining a large collection of methods to identify potential miRNAs in complex diseases.

A significant concern regarding the computational approaches used in this study is controlling false positives from both public databases and prediction results caused by computational tools. In our framework, to minimize the effect of these false positives, we first performed a comprehensive compilation from multiple data sources to identify genes and miRNAs relevant to GBM. Next, we chose the most popular databases and software to conduct the prediction. Finally, we applied stringent parameters in the prediction of TF-gene/miRNA, miRNA-gene/TF, and gene-gene relationships. For TF-gene/miRNA and miRNA-gene/TF, we further required conservation among multiple mammalian genomes. Thus, our framework could potentially detect the most important regulatory relationships and might be applied to other complex diseases for the purpose of deciphering their regulatory systems and identifying critical miRNAs.

Compared to high-throughput and low-throughput experimental methods that have been used to discover and profile miRNAs, our computational framework could complement them and facilitate the discovery of critical miRNAs in the pathology of disorders. As much more regulatory data is expected to be released in the near future, such as ChIP-Seq (chromatin immunoprecipitation sequencing), RNA-Seq (transcriptome sequencing) and GRO-Seq (global run-on sequencing), this framework could be improved with the integration of high-throughput data by filtering out interactions in low confidence.

One important output of this comprehensive study is the GBM-specific miRNA-TF combinatory regulatory network. The regulatory network was massive and complex, presenting us with another challenging task: finding the tactic to decipher this huge network to mine the important regulatory components. Recently, pathway analysis has been reported as a useful approach to investigate the pathology of complex diseases [6], [58]. Specifically in our work, our strategy was to apportion the large regulatory network and extract relatively small but functionally critical subnetworks for pathways that have been previously implicated in the corresponding disease. We then performed network topology analyses and investigated modularity to identify critical miRNAs in these small subnetworks.

To demonstrate this strategy, we used the Notch signaling pathway as an example and found six critical miRNAs in the pathway in GBM (Figure 4). Among them, miR-34a has already been shown in an independent study led by one of the authors in 2009 (B.P.) to be down-regulated in GBM, target Notch family members, and cause differentiation in GBM stem-like cells [59], [60]. Additional studies have shown that this miRNA has been involved in the Notch pathway in other cancers such as medulloblastoma [61], pancreatic cancer [62] and carcinoma [63]. Moreover, miR-124 and miR-137 have functioned in a tumor-suppressive fashion in GBM and caused differentiation when re-expressed in GBM cells [24]. miR-9 has also been strongly linked to GBM subtypes in a recent analysis [25]. Interestingly, miR-124 has been reported to be involved in the Notch signaling pathway during *Ciona intestinalis* neuronal development [64]. The evidence from these studies suggests the effectiveness of our approach. Further experimental validation of these miRNAs is warranted.

Among the six miRNAs, the most noteworthy one is miR-34a. It regulates a number of target proteins that are involved in cell cycle, apoptosis, differentiation and cellular development [65]. In the independent study mentioned above, led by one of the authors (B.P.), the effects of miR-34a on *MET*, *NOTCH1*, *NOTCH2*, *CDK6*, and *PDGFRA* expression in brain tumor cells and stem cells were tested. The results showed that miR-34a suppressed brain tumor growth by targeting *MET* and *Notch* [66]. To check if these results exist in our predicted regulatory network, we further extracted miR-34a FFLs and merged them to form a miR-34a-specific regulatory network (Figure S9). Among 15 miR-34a targets, 8 (*NOTCH2*, *MET*, *PDGFRA*, *JAG1*, *MYCN*, *BCL2*, *DCX*, and *CACNA1E*) belonged to GMB-related genes and 7 (*FOSB*, *FOSL1*, *NFE2L1*, *NR4A2*, *SMAD4*, *TCF12*, and *YY1*) belonged to human TFs. Among the 8 GBM-related genes, *NOTCH2* and *MET* have been reported in our previous study to be targeted by miR-34a, while *PDGFRA* was not [66]. *JAG1* has been reported to be targeted by the miRNA in the regulation of human monocyte-derived dendritic cell differentiation [67]. *MYCN* has been reported to be targeted by miR-34a in neuroblastoma cells [68], [69] and somatic cell reprogramming [70]. *BCL2* has been reported to be targeted by the same miRNA in neuroblastoma cells [69]. All 7 targeted TFs were significantly involved in the transcription of DNA according to Biology Function Analysis in IPA (Ingenuity Pathway Analysis) (Fisher's exact test, *P*-value = 8.75×10^−9^) as expected. Among them, *YY1* has been reported to be directly targeted by miR-34a in neuroblastoma cells [71]. Taken together, miR-34a is likely not only regulates GBM-related genes directly but also regulates the TFs for gene expression through transcriptional mechanism. This assertion needs further experimental confirmation. While our analyses, especially of miR-34a and its targets, support the utility of our regulatory network framework, it still needs to be improved. Most GBM-related genes have not been confirmed to be causal, the human TF and miRNA binding profiles are neither complete nor error- or bias-free, and reverse engineering software has its own weaknesses.

This work represents the first application of a 4-node FFL as a regulatory motif in complex disease. Although there have been several genome-wide studies applying integrative regulation of TFs and miRNAs [30], [72], [73], none have considered gene coexpression profiles in an FFL model. The 4-node FFL model contains four components: one miRNA, one TF, and two co-expressed genes related to GBM (Figure 2). There are four types of possible regulations between the co-expressed genes and the TF and miRNA, making the regulatory network more informative and tolerant (Figure S10). Compared with 3-node FFLs, the main impact of 4-node FFLs is the recruitment of more GBM-related genes and regulatory relationships into the regulatory network (Table 2, Figure S3, and Figure S4). We found that 4-node FFLs tended to regulate the genes that might belong to the same biological processes, the same protein family, or be located in the same cellular components (Figure S2). Additionally, among the 20 GBM-related genes involved in the miR-34a-specific regulatory network, 3 were in the 3-node FFLs and 4-node FFLs, 11 from 4-node FFLs, and 6 from 3-node FFLs. This observation indicated that the recruitment of GBM-related genes in miR-34a network was greatly improved by applying the 4-node FFLs. In summary, our comparison of the 4-node and 3-node FFLs and the performance in the recruitment of GBM-related genes by the 3-node FFLs and 4-node FFLs to the miR-34a-specific regulatory network indicate that both are useful models, and they may complement each other in a regulatory network analysis.

Another interesting observation in this study is composite-FFLs, in which TF and miRNA regulate each other. The regulation between a TF and a miRNA has been defined as a TF↔miRNA feedback loop [38]. In our study, we observed 40 TF↔miRNA feedback loops in 3-node FFLs and 24 TF↔miRNA feedback loops in 4-node FFLs. Among the two sets of feedback loops, there were 19 loops in common between two sets, resulting in 45 unique feedback loops in the whole regulatory network for GBM. Compared to the 759 unique TF-miRNA regulatory relationships and the 505 miRNA-TF regulatory relationships in the regulatory network, the TF↔miRNA regulatory relationships were rarely observed. This low frequency is consistent with previous reports involving a pure transcriptional regulatory network [42]. However, interestingly, these TF↔miRNA feedback loops regulate 101 GBM-related genes, accounting for 57.38% of the GBM-related genes (176) in the GBM miRNA-TF mediated regulatory network. This observation indicated that composite-FFLs are more effective in unveiling the regulatory systems underlying the complex disease.

## Materials and Methods

### Genes and miRNAs related to GBM

To collect genes involved in the pathology of GBM, we compiled GBM-related genes from six sources, which included multiple types of variations with experimental evidence, such as point mutation, gene fusion, structure rearrangement, and copy number variation. These sources included the Catalogue Of Somatic Mutations In Cancer (COSMIC, version 51) [74], the Online Mendelian Inheritance in Man (OMIM) [75], The Cancer Genome Atlas (TCGA) [6], and the Genetic Association Database (GAD) [76], as well as one recently published integrative genomic analysis of GBM [39] and two genome-wide association studies [4], [5] (Text S1). We mapped these genes to Entrez gene symbols and ultimately obtained 415 unique genes.

To collect a set of dysregulated miRNAs in GBM, we conducted a comprehensive literature search to identify studies that directly assess miRNA dysregulation in GBM patients' cell lines or tissues. We first searched the miR2Disease [77], PhenomiR [78] and HMDD [79] databases for relevant articles using the keyword “glioblastoma” and PubMed using the keywords “glioblastoma AND microRNA.” Then, we manually checked each title and abstract for relevance and reviewed the full text if the abstract indicated that the article reported associations between miRNA expression and GBM. As a result, we included 24 papers that directly assessed miRNA expression in GBM samples or cell lines. From these papers, we retrieved 134 miRNAs with up/down-regulated information, which were mapped to 124 unique mature miRNAs based on human miRNAs from miRBase [80].

### Prediction of posttranscriptional repression of miRNA to gene/TF (miRNA- gene/TF)

Currently, several online databases that predict binding sites and target genes of individual miRNAs are available, such as PicTar [81], TargetScan [35], [82], and miRanda [83]. Among them, TargetScan has demonstrated the best performance compared to other miRNA target prediction software [84], [85]. Therefore, we extracted the miRNA-gene pairs between GBM-related miRNAs and GBM-related genes from the TargetScan server (version 5.2, February 2011) [35]. We required that miRNA-target interactions be evolutionarily conserved in four species (human, mouse, rat and dog) and have a total context score higher than −0.30 [86]. The score quantitatively measures the overall target efficacy [84], [85]. To obtain the posttranscriptional repression of miRNAs on TFs, we first retrieved 428 TFs that have human genes as targets from the TRANSFAC Professional database (release 2011.4) [34] and used the same procedure to obtain the relationships between miRNAs and TFs.

### Prediction of regulatory relationship between TF and gene/miRNA (TF-gene/miRNA)

To predict the regulatory relationship between TF and gene/miRNA, we first downloaded the defined promoter region (−1500/+500 around TSS) of 415 GBM-related genes or 134 GBM-related miRNAs from the UCSC Table Browser [87]. Then, we performed a binding sites search using the Match™ software that is integrated in TRANSFAC Professional (release 2011.4) [36]. For the purpose of this study, we used pre-calculated cut-offs to minimize false positive matches (minFP) and create a high-quality matrix. To restrict the search, we required a core score of 1.00, a matrix score of 0.95, and TFs that only belong to the human genome. To further reduce false positive prediction, we required the predicted pairs to be conserved among humans, mice and rats [73].

### Calculation of co-regulated genes (gene-gene)

Recently, Verhaak et al. [39] integrated the gene expression data from 200 GBM and two normal brain samples examined by three gene expression microarray platforms (Affymetrix HuEx array, Affymetrix U133A array, and Agilent 244 K array) into a single, unified data set of 11,861 genes using a factor analysis model. Then, they filtered the unified genes down to 1,740 genes with consistent but highly variable expression across the platforms using several filters to eliminate unreliably measured genes. We directly applied the resulting data to identify co-regulated genes. Among the 415 GBM-related genes we collected, 120 were included in the 1,740 genes. We estimated co-regulated relationships among these genes via the ARACNE software, which implemented the mutual information (MI) theory to identify transcriptional interactions between genes [40]. We used a high significance threshold for MI values with a *P*-value of 1.0×10^−7^ to sort out possible false positive and true negative data. To remove indirect regulatory relationship, we employed a data process inequality (DPI) tolerance of 0.15 according to the recommendation by Margolin et al. [88].

### Significant miRNA-TF co-occurring pairs

To identify TF and miRNA pairs that cooperatively regulate the same target genes, we calculated a *P*-value using a cumulative hypergeometric test [43] based on the common targets of any pair of miRNAs and TFs as in the following function:
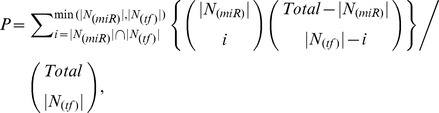
where 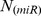 is the number of genes targeted by a given miRNA, 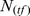 is the number of genes regulated by a given TF, and *Total* is the number of common genes between all human genes targeted by human miRNAs and all human genes regulated by all human TFs. We further used the false discovery rate (FDR) to adjust for multiple testing [89], and only those pairs with a corrected *P*-value less than 0.05 were chosen as significant pairs of regulators.

### Calculation of Gene Ontology semantic similarity

To quantify functional similarity, we calculated GO semantic similarity scores for the GO terms for each pair of the co-regulated genes using the R GOSemSim package [44]. For each of the three GO categories (BP: biological process, MF: molecular function, and CC: cellular component), the semantic similarity scores were computed for all gene pairs in the 3-node and 4-node FFLs. A gene pair was compiled from any two genes targeted by the same miRNA-TF pairs. To evaluate the statistical significance of the functional similarity of co-targeted genes in FFLs, we randomly selected the same number of genes in 3-node or 4-node FFLs from the 20,441 Entrez protein-coding genes with GO annotations, and calculated their GO similarities. We repeated this process 1,000 times. We performed a Kolmogorov-Smirnov test (KS-test) to examine whether the GO similarity of all the gene pairs from the FFLs is significantly greater than that of randomly selected pairs.

### Network and subnetwork generation, analyses, and functional evaluation

In this work, we constructed three major networks. The first network was the GBM-specific miRNA-TF mediated gene regulatory network, which was generated by converging all significant 3-node and 4-node FFLs. The second one was the GBM composite regulatory network generated by merging only those significant 3-node and 4-node composite-FFLs. The third one was the subnetwork for the Notch signaling pathway. We first collected the genes belonging to the Notch pathway from the KEGG and Ingenuity systems and merged those FFLs that included at least one Notch pathway gene to generate a Notch-specific regulatory network in GBM.

Considering the complexity of regulatory networks and our goal of distilling critical elements, we simplified the network analysis by disregarding the direction of the edges. We computed nodes' degrees and their distributions in order to assess network characteristics. The degree of a node, the network's most elementary characteristic, is measured by the number of links of the node in the network. If the degree distribution of one network follows a power law, the network would have only a small portion of nodes with a large number of links (i.e., hubs) [47]. To determine the hubs in our network, we applied the method proposed by Yu et al. [50] to draw a degree distribution for each node in the network. For local network analysis, we used the software CFinder (version 2.0.5) [57] to generate tightly connected sub-networks from the pathway network, and we then visualized them using Cytoscape (version 2.8) [90].

To identify pathways overrepresented in GBM-related genes from the GBM composite regulatory network, we performed a pathway enrichment analysis using the Core Analysis Tool in Ingenuity Pathway Analyses (IPA) from Ingenuity Systems [68]. Given a list of genes, a right-tailed Fisher's exact test was performed for the enrichment of these genes based on its hand-curated canonical pathway database. To control the error rate in the analysis results, IPA also provided a corrected *P*-value based on the Benjamini-Hochberg method [89]. GO and KEGG enrichment of the subnetworks was analyzed using WebGestalt [46].

## Supporting Information

Figure S1 **A catalogue of merged feed-forward regulatory loops (FFLs).** Each composed of a known transcription factor (TF), a mature microRNA (miRNA) and a list of GBM-related genes or a list of GBM-related co-regulated gene pairs. According to the relationship between the transcription factor (TF) and microRNA (miRNA), the mixed FFLs were classified as the TF-FFL model (the TF directly regulates the miRNA), miRNA-FFL model (the miRNA only directly regulates the TF) or composite-FFL model (the TF and the miRNA regulate each other). The relationships represented by solid lines are required while the relationships represented by dot lines are not required.
(TIF)

Figure S2 **Cumulative distributions of functional semantic scores for biological process (BP), molecular function (MF), and cellular component (CC) of gene pairs for randomly selected genes (black), co-regulated genes in 3-node FFLs (blue) and co-regulated genes in 4-node FFLs (red).** The inserted *P*-values were calculated by the Kolmogorov-Smirnov test.
(TIF)

Figure S3 **Intersects of GBM-related genes, GBM-related miRNAs and TFs from TF-FFLs, miRNA-FFLs and composite-FFLs in 3-node model (A) and 4-node model (B), respectively.**
(TIF)

Figure S4 **Intersects of nodes (A) and links (B) in 3-node FFLs and those in 4-node FFLs.**
(TIF)

Figure S5 **Degree distributions of all nodes in GBM-specific miRNA-TF mediated regulatory network.** The red for GBM-related microRNAs, green dots are for GBM-related genes, and blue for TFs. The Y-axis represents the proportion of nodes having a specific degree.
(TIF)

Figure S6 **Notch-specific miRNA-TF mediated regulatory subnetworks specific for GBM identified by software CFinder.** Different subnetworks are shown by IDs from ‘A’ to ‘D’. Nodes in red (round rectangle) correspond to GBM-related miRNAs, green ones (ellipse) correspond to GBM-related genes, and blue ones (triangle) correspond to transcription factors (TFs). The edge colors represent the different relation: red represents the repression of miRNAs to genes or TFs, and blue represents the regulation of TFs to genes or miRNAs.
(TIF)

Figure S7 **Comparison between the GBM Notch-specific regulatory network (A) and the relative network after removing the centered subnetwork (B).** Nodes in red (round rectangle) correspond to GBM-related miRNAs, green ones (ellipse) correspond to GBM-related genes, and blue ones (triangle) correspond to transcription factors (TFs). Among them, nodes in yellow are centred nodes in the network. The edge colors represent the different relation: red represents the repression of miRNAs to genes or TFs, and blue represents the regulation of TFs to genes or miRNAs.
(TIF)

Figure S8 **Comparison between the GBM Notch-specific miRNA-TF regulatory network (A) and the relative network after removing the centred subnetwork and its directly linked nodes (B).** Nodes in red (round rectangle) correspond to GBM-related miRNAs, green ones (ellipse) correspond to GBM-related genes, and blue ones (triangle) correspond to transcription factors (TFs). Among them, nodes in yellow are centered nodes and their directly interacting nodes in the network. The edge colors represent the different relation: red represents the repression of miRNAs to genes or TFs, and blue represents the regulation of TFs to genes or miRNAs.
(TIF)

Figure S9 **miR-34a Specific regulatory network extracted from GBM-specific miRNA-TF mediated regulatory network.** One node in red (round rectangle) corresponds to one GBM-related miRNA (has-miR-34a), green nodes (ellipse) correspond to GBM-related genes, and blue ones (triangle) correspond to transcription factors (TFs). The edge colors represent the different relation: red represents the repression of miRNAs to genes or TFs, and blue represents the regulation of TFs to genes or miRNAs.
(TIF)

Figure S10 **Four types of regulation between coexpressed genes and two regulatory elements: TF and miRNA.** The relationships represented by solid lines are required. Among the two relationships by dash dot lines, at least one is required. Nodes in orange (round rectangle) correspond to GBM-related miRNAs, red ones (ellipse) correspond to GBM-related genes, and blue ones (triangle) correspond to transcription factors (TFs).
(TIF)

Table S1 **Six sources for collection of glioblastoma (GBM)-related genes.**
(DOC)

Table S2 **Merged 3-node FFLs including TF-FFLs, miRNA-FFLs and composite-FFLs.**
(XLS)

Table S3 **Comparison of number of targets with same protein family annotation in FFLs with randomly selected genes.**
(DOC)

Table S4 **Pathways significantly enriched for 153 GBM-related genes in 3-node FFLs.**
(DOC)

Table S5 **Merged 4-node FFLs including TF-FFLs, miRNA-FFLs and composite-FFLs.**
(XLS)

Table S6 **Significantly enriched KEGG pathways in the 176 genes.**
(DOC)

Table S7 **GBM-specific miRNA-TF mediated regulatory network.**
(XLS)

Table S8 **miRNAs potentially involved in GBM-specific Notch signaling pathway.**
(DOC)

Text S1 **Compiling glioblastoma-related genes (GBM-related genes) from multiple datasets.**
(DOC)

Text S2 **Pfam annotation used to test whether the target protein in each merged FFL tend to belong to same protein family.**
(DOC)
