# Supplementary figures and images for: Uncovering MicroRNA and Transcription Factor Mediated Regulatory Networks in Glioblastoma
Source: PLoS Comput Biol. 2012 Jul 19;8(7):e1002488. doi: 10.1371/journal.pcbi.1002488 (PMC3400583; doi:10.1371/journal.pcbi.1002488)

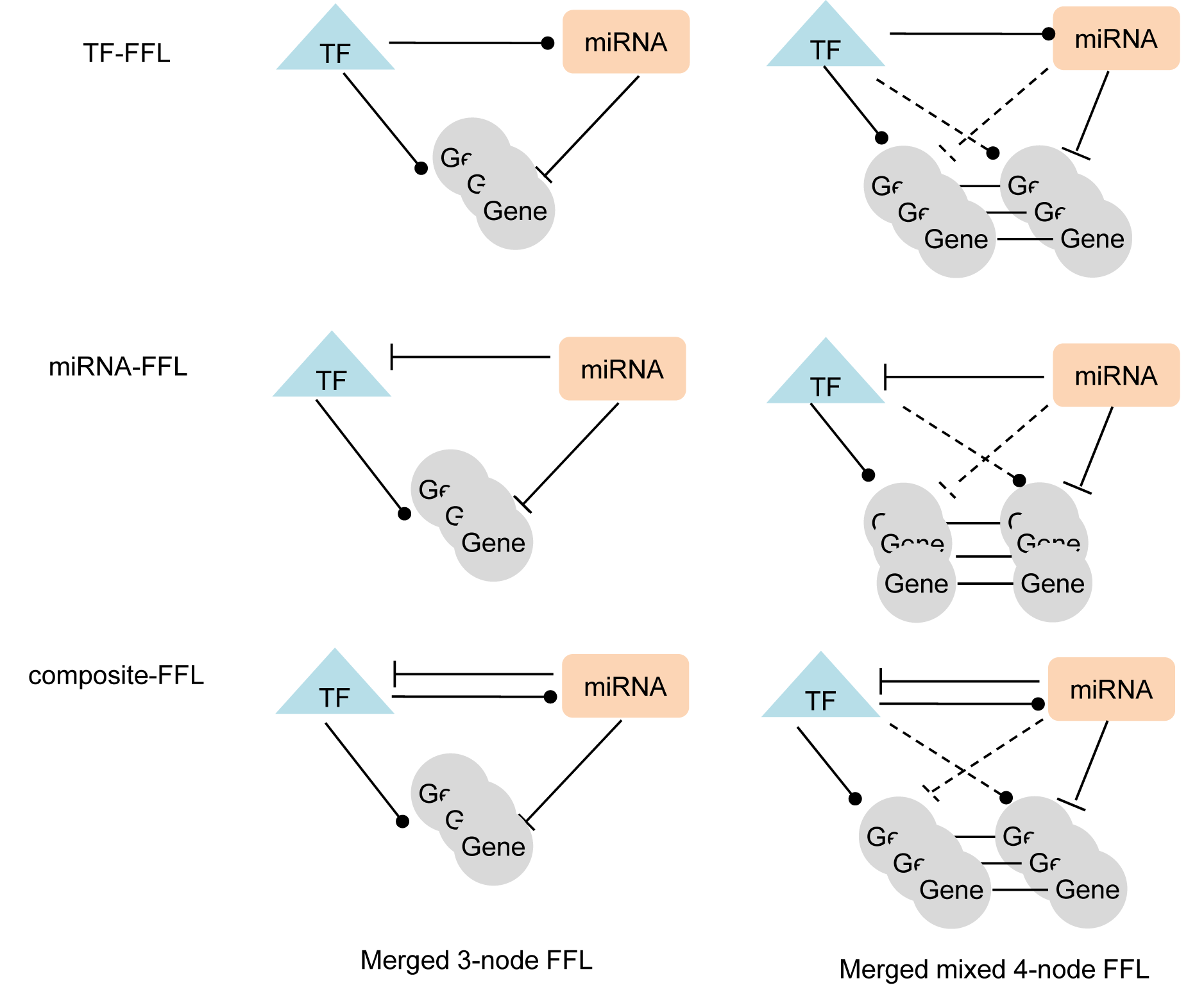

Supplement: Figure S1 — A catalogue of merged feed-forward regulatory loops (FFLs). Each composed of a known transcription factor (TF), a mature microRNA (miRNA) and a list of GBM-related genes or a list of GBM-related co-regulated gene pairs. According to the relationship between the transcription factor (TF) and microRNA (miRNA), the mixed FFLs were classified as the TF-FFL model (the TF directly regulates the miRNA), miRNA-FFL model (the miRNA only directly regulates the TF) or composite-FFL model (the TF and the miRNA regulate each other). The relationships represented by solid lines are required while the relationships represented by dot lines are not required. (TIF) [file pcbi.1002488.s001.tif]

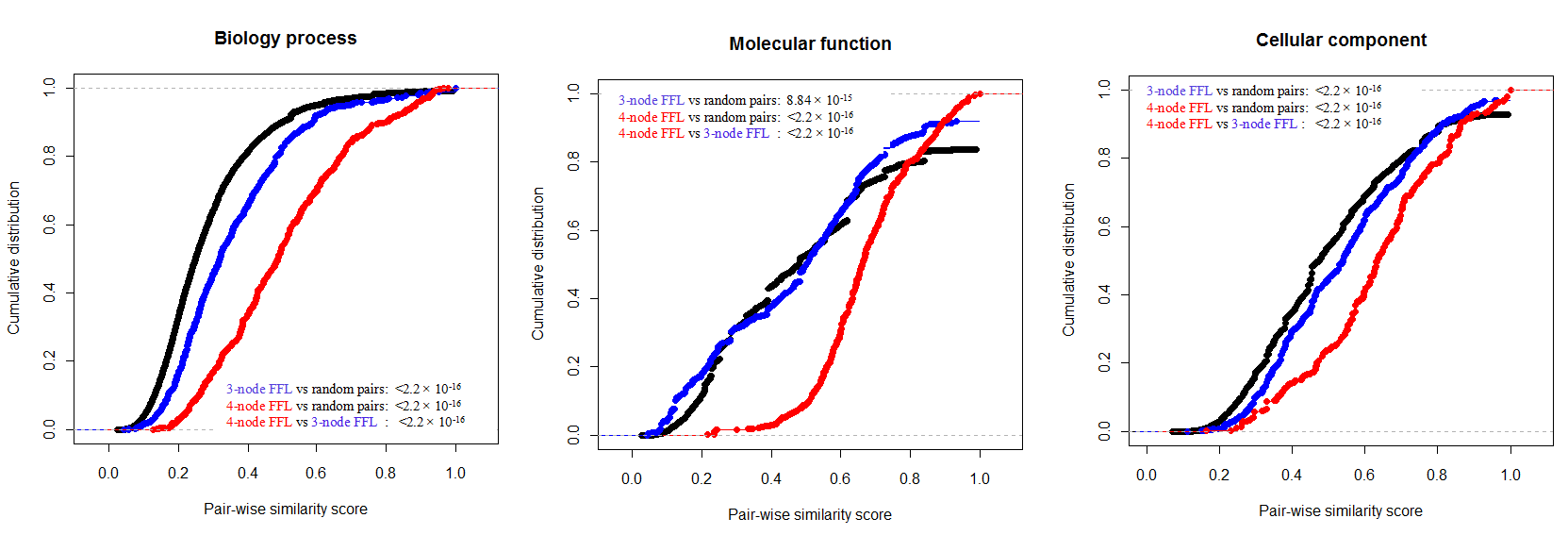

Supplement: Figure S2 — Cumulative distributions of functional semantic scores for biological process (BP), molecular function (MF), and cellular component (CC) of gene pairs for randomly selected genes (black), co-regulated genes in 3-node FFLs (blue) and co-regulated genes in 4-node FFLs (red). The inserted P-values were calculated by the Kolmogorov-Smirnov test. (TIF) [file pcbi.1002488.s002.tif]

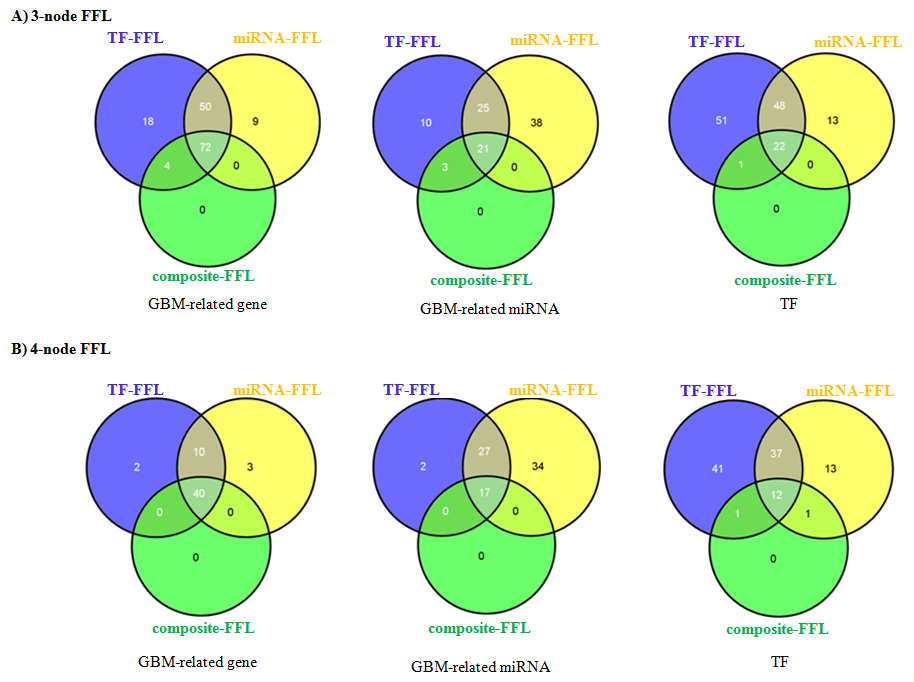

Supplement: Figure S3 — Intersects of GBM-related genes, GBM-related miRNAs and TFs from TF-FFLs, miRNA-FFLs and composite-FFLs in 3-node model (A) and 4-node model (B), respectively. (TIF) [file pcbi.1002488.s003.tif]

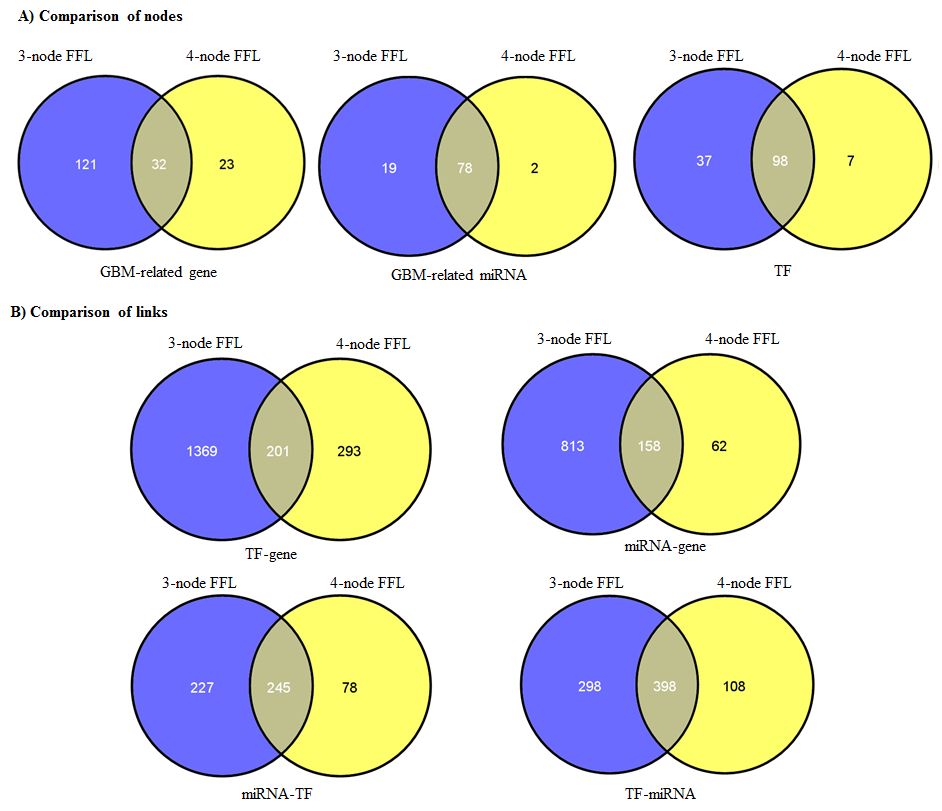

Supplement: Figure S4 — Intersects of nodes (A) and links (B) in 3-node FFLs and those in 4-node FFLs. (TIF) [file pcbi.1002488.s004.tif]

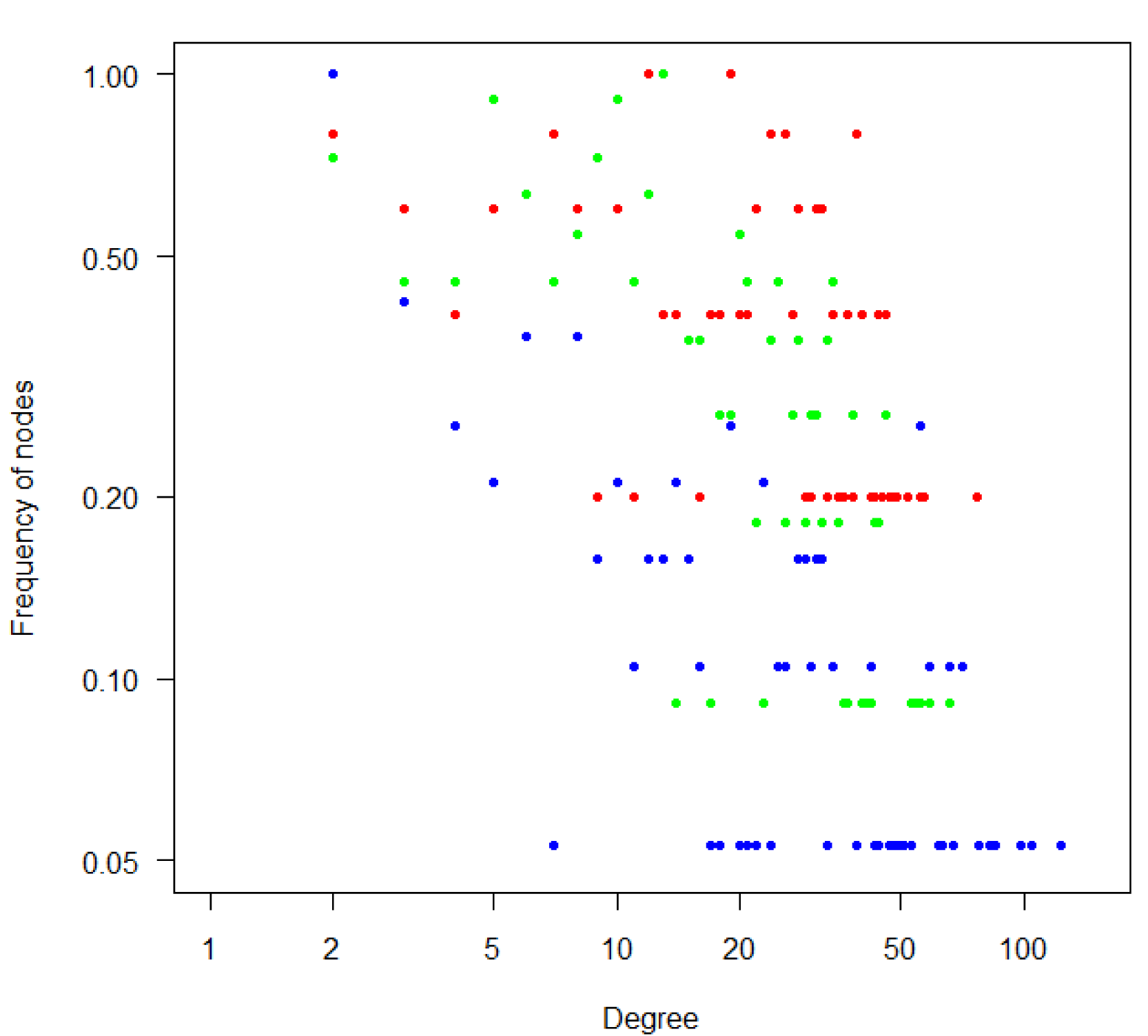

Supplement: Figure S5 — Degree distributions of all nodes in GBM-specific miRNA-TF mediated regulatory network. The red for GBM-related microRNAs, green dots are for GBM-related genes, and blue for TFs. The Y-axis represents the proportion of nodes having a specific degree. (TIF) [file pcbi.1002488.s005.tif]

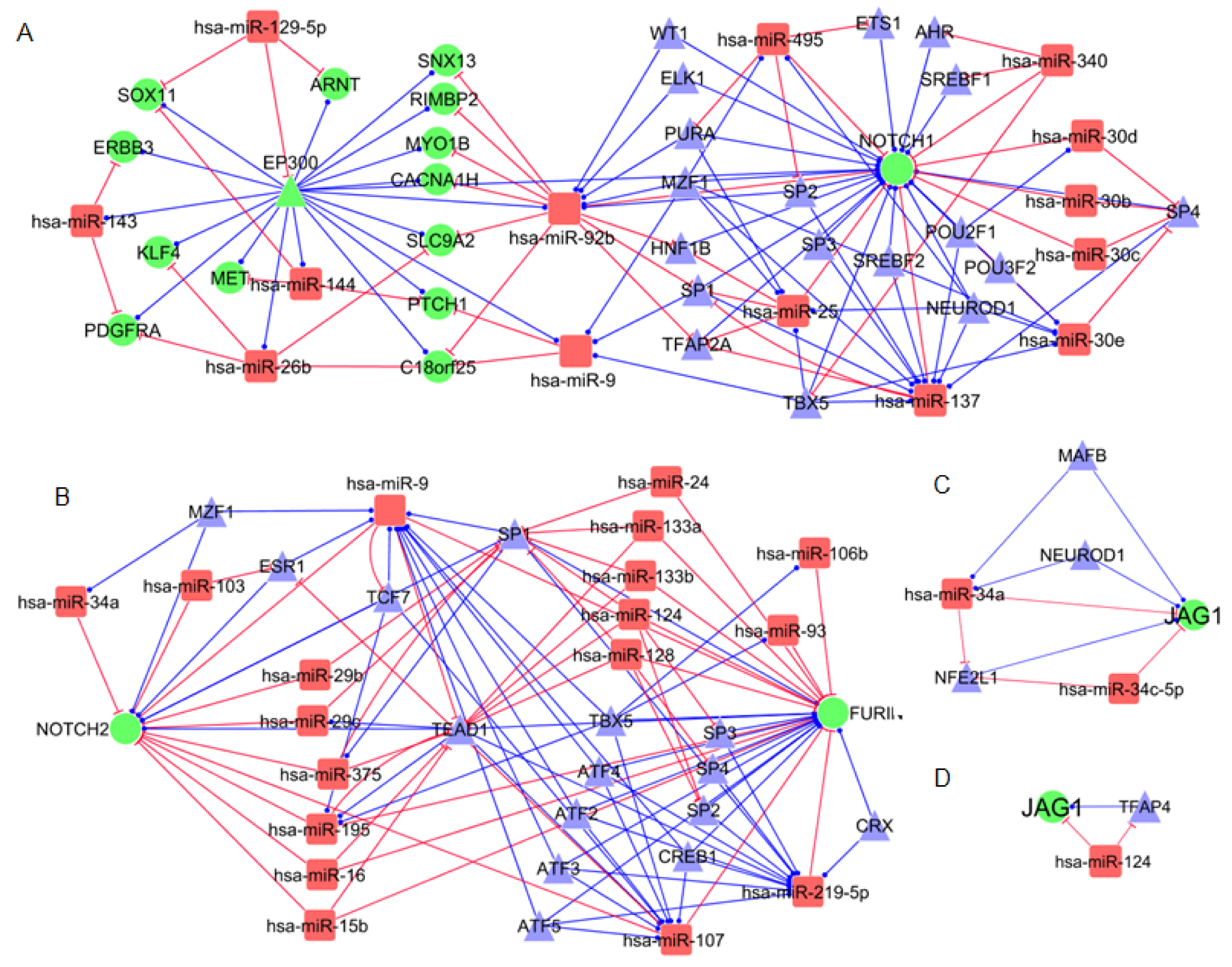

Supplement: Figure S6 — Notch-specific miRNA-TF mediated regulatory subnetworks specific for GBM identified by software CFinder. Different subnetworks are shown by IDs from ‘A’ to ‘D’. Nodes in red (round rectangle) correspond to GBM-related miRNAs, green ones (ellipse) correspond to GBM-related genes, and blue ones (triangle) correspond to transcription factors (TFs). The edge colors represent the different relation: red represents the repression of miRNAs to genes or TFs, and blue represents the regulation of TFs to genes or miRNAs. (TIF) [file pcbi.1002488.s006.tif]

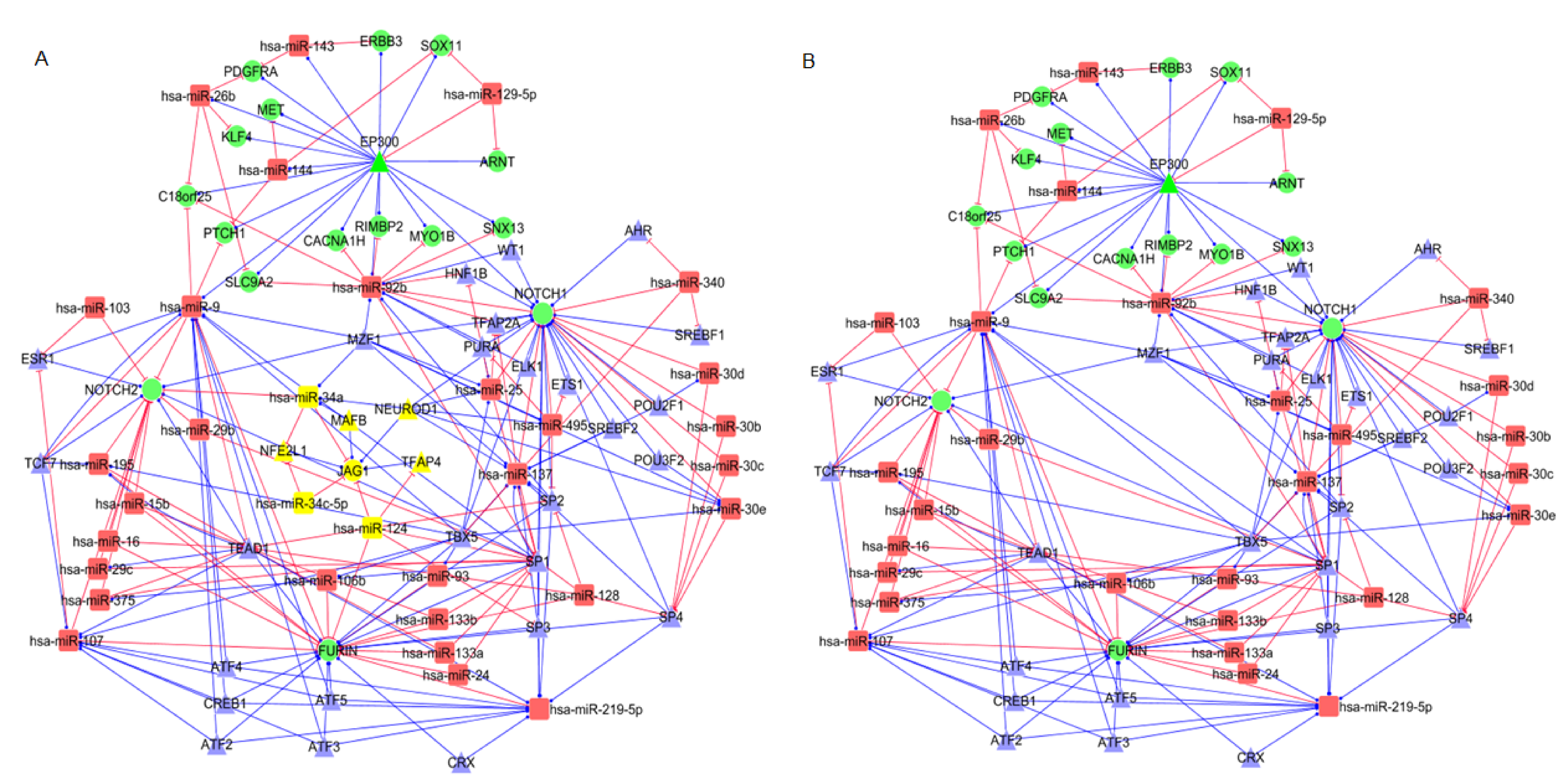

Supplement: Figure S7 — Comparison between the GBM Notch-specific regulatory network (A) and the relative network after removing the centered subnetwork (B). Nodes in red (round rectangle) correspond to GBM-related miRNAs, green ones (ellipse) correspond to GBM-related genes, and blue ones (triangle) correspond to transcription factors (TFs). Among them, nodes in yellow are centred nodes in the network. The edge colors represent the different relation: red represents the repression of miRNAs to genes or TFs, and blue represents the regulation of TFs to genes or miRNAs. (TIF) [file pcbi.1002488.s007.tif]

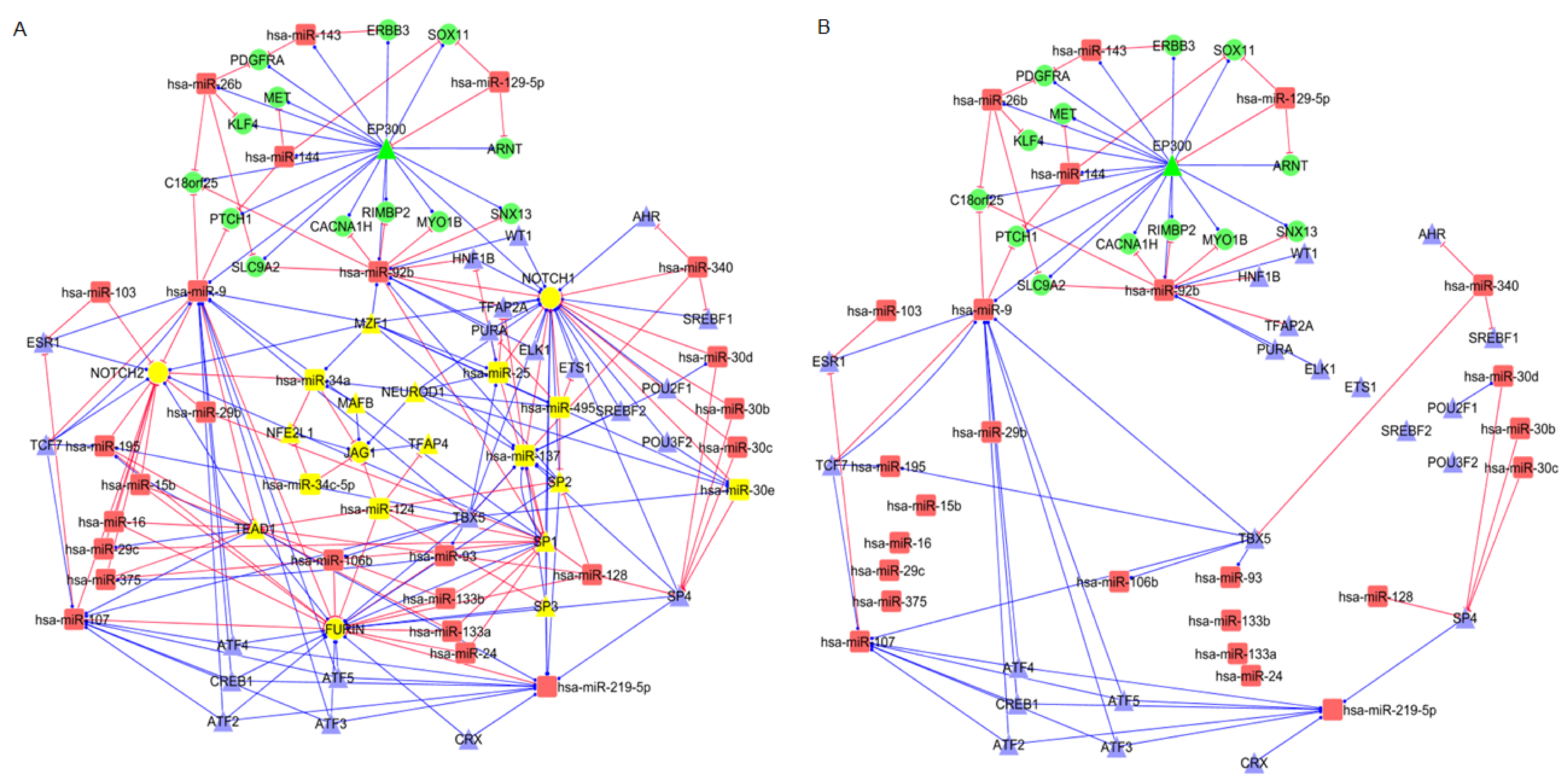

Supplement: Figure S8 — Comparison between the GBM Notch-specific miRNA-TF regulatory network (A) and the relative network after removing the centred subnetwork and its directly linked nodes (B). Nodes in red (round rectangle) correspond to GBM-related miRNAs, green ones (ellipse) correspond to GBM-related genes, and blue ones (triangle) correspond to transcription factors (TFs). Among them, nodes in yellow are centered nodes and their directly interacting nodes in the network. The edge colors represent the different relation: red represents the repression of miRNAs to genes or TFs, and blue represents the regulation of TFs to genes or miRNAs. (TIF) [file pcbi.1002488.s008.tif]

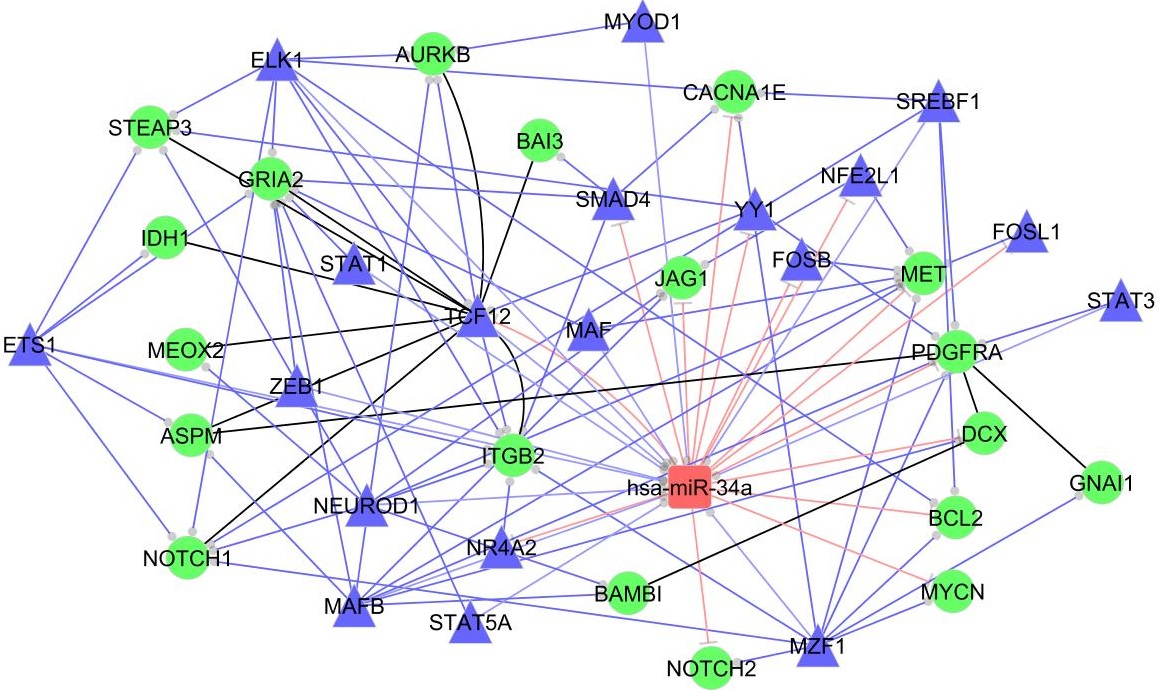

Supplement: Figure S9 — miR-34a Specific regulatory network extracted from GBM-specific miRNA-TF mediated regulatory network. One node in red (round rectangle) corresponds to one GBM-related miRNA (has-miR-34a), green nodes (ellipse) correspond to GBM-related genes, and blue ones (triangle) correspond to transcription factors (TFs). The edge colors represent the different relation: red represents the repression of miRNAs to genes or TFs, and blue represents the regulation of TFs to genes or miRNAs. (TIF) [file pcbi.1002488.s009.tif]

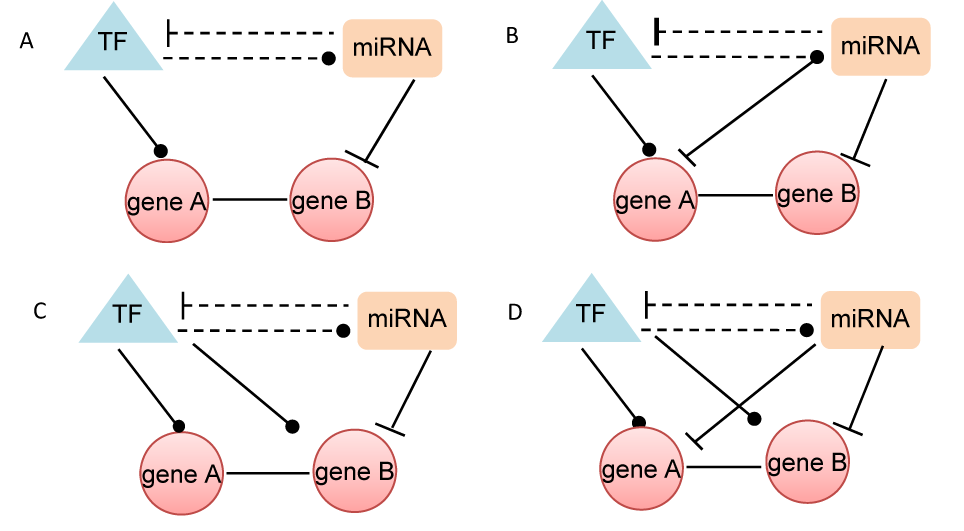

Supplement: Figure S10 — Four types of regulation between coexpressed genes and two regulatory elements: TF and miRNA. The relationships represented by solid lines are required. Among the two relationships by dash dot lines, at least one is required. Nodes in orange (round rectangle) correspond to GBM-related miRNAs, red ones (ellipse) correspond to GBM-related genes, and blue ones (triangle) correspond to transcription factors (TFs). (TIF) [file pcbi.1002488.s010.tif]
