# Supplementary material for: Uncovering MicroRNA and Transcription Factor Mediated Regulatory Networks in Glioblastoma
Source: PLoS Comput Biol. 2012 Jul 19;8(7):e1002488. doi: 10.1371/journal.pcbi.1002488 (PMC3400583; doi:10.1371/journal.pcbi.1002488)
Supplement: Table S1 — Six sources for collection of glioblastoma (GBM)-related genes. (DOC) [file pcbi.1002488.s011.doc]

**Table S1. Six data sources for collection of glioblastoma (GBM)-related genes.**

| **Data sources** | **Web link** | **Number of GBM genes** | **Note** |
| --- | --- | --- | --- |
| COSMIC | http://www.sanger.ac.uk/genetics/CGP/cosmic/ | 179 | version 51, February 7, 2011 |
| TCGA | http://tcga-data.nci.nih.gov/docs/somatic_mutations/tcga_mutations.htm | 223 | TCGA (2008) |
| GAD | http://geneticassociationdb.nih.gov/ | 6 | February 28, 2011 |
| OMIM | http://www.ncbi.nlm.nih.gov/entrez/query.fcgi?db=OMIM | 3 | February 28, 2011 |
| Parson et al [2008] | http://www.ncbi.nlm.nih.gov/pubmed/20129251 | 42 | *CAN*-genes^a^ |
| GWAS | http://www.ncbi.nlm.nih.gov/pubmed/19578366 | 6 | Wrensch et al. [2009] and Shete et al. [2009] |

^a^ *CAN*-genes were GBM candidate cancer genes that were obtained by integrating the mutational data from sequencing and analysis of copy number alternations.
