# Supplementary material for: Uncovering MicroRNA and Transcription Factor Mediated Regulatory Networks in Glioblastoma
Source: PLoS Comput Biol. 2012 Jul 19;8(7):e1002488. doi: 10.1371/journal.pcbi.1002488 (PMC3400583; doi:10.1371/journal.pcbi.1002488)
Supplement: Table S3 — Comparison of number of targets with same protein family annotation in FFLs with randomly selected genes. (DOC) [file pcbi.1002488.s013.doc]

**Table S3. Comparison of number of targets with same protein family annotation in FFLs with randomly selected genes.**

| **FFL**^a^ | **Number of merged FFLs** | **Number of merged FFLs with at least 2 targets** | **Number of target pairs in same FFL** | **Number of pairs sharing Pfam^b^ annotation** | **Number of pairs not sharing Pfam annotation** | **Fisher's exact test *P*-value^b^** |
| --- | --- | --- | --- | --- | --- | --- |
| 3-node FFL |  |  |  |  |  |  |
| TF-FFL | 656 | 499 | 3,450 | 166 | 3,284 | < 2.2 × 10^-16^ |
| miRNA-FFL | 432 | 304 | 2,138 | 70 | 2,068 | < 2.2 × 10^-16^ |
| composite-FFL | 40 | 30 | 664 | 16 | 648 | 0.0003 |
| All 3-node | 1,128 | 833 | 6,252 | 252 | 6,000 | < 2.2 × 10^-16^ |
| 4-node FFL |  |  |  |  |  |  |
| TF-FFL | 482 | 291 | 1,812 | 536 | 1,276 | < 2.2 × 10^-16^ |
| miRNA-FFL | 299 | 154 | 694 | 430 | 264 | < 2.2 × 10^-16^ |
| Composite-FFL | 24 | 17 | 202 | 116 | 86 | < 2.2 × 10^-16^ |
| All 4-node | 805 | 462 | 2,708 | 1,082 | 1,626 | < 2.2 × 10^-16^ |
| 1,000 times randomly selected genes |  |  |  | 18,736 | 2,156,864 |  |

^a^FFL: feed-forward loop.

^b^Pfam: protein family annotation database.
