# Supplementary material for: Uncovering MicroRNA and Transcription Factor Mediated Regulatory Networks in Glioblastoma
Source: PLoS Comput Biol. 2012 Jul 19;8(7):e1002488. doi: 10.1371/journal.pcbi.1002488 (PMC3400583; doi:10.1371/journal.pcbi.1002488)
Supplement: Table S4 — Pathways significantly enriched for 153 GBM-related genes in 3-node FFLs. (DOC) [file pcbi.1002488.s014.doc]

**Table S4.** Pathways significantly enriched for 153 GBM-related genes in 3-node FFLs.

| **Ranking** | **Pathway** | **Adjusted *P*-value^a^** |
| --- | --- | --- |
| 1 | Pathways in cancer^b^ | 9.27 × 10^-25^ |
| 2 | Focal adhesion^b^ | 3.06 × 10^-16^ |
| 3 | Prostate cancer^b^ | 4.01 × 10^-15^ |
| 4 | Melanoma^b^ | 1.09 × 10^-14^ |
| 5 | Neurotrophin signaling pathway^b^ | 1.63 × 10^-13^ |
| 6 | Glioma^b^ | 1.63 × 10^-13^ |
| 7 | MAPK signaling pathway^b^ | 1.85 × 10^-13^ |
| 8 | Colorectal cancer^b^ | 1.78 × 10^-12^ |
| 9 | Non-small cell lung cancer | 6.40 × 10^-11^ |
| 10 | Renal cell carcinoma^b^ | 4.99 × 10^-10^ |
| 11 | Pancreatic cancer | 5.69 × 10^-10^ |
| 12 | Chronic myeloid leukemia^b^ | 7.31 × 10^-10^ |
| 13 | Endometrial cancer | 1.64 × 10^-9^ |
| 14 | Small cell lung cancer | 1.64 × 10^-9^ |
| 15 | Regulation of actin cytoskeleton | 1.16 × 10^-8^ |
| 16 | Fc epsilon RI signaling pathway | 2.90 × 10^-8^ |
| 17 | Type II diabetes mellitus | 3.39 × 10^-8^ |
| 18 | ECM-receptor interaction | 3.95 × 10^-8^ |
| 19 | Acute myeloid leukemia | 1.36 × 10^-7^ |
| 20 | Adherens junction | 5.85 × 10^-7^ |
| 21 | Cell cycle | 6.16 × 10^-7^ |
| 22 | Axon guidance^b^ | 6.16 × 10^-7^ |
| 23 | Bladder cancer | 6.16 × 10^-7^ |
| 24 | Cytokine-cytokine receptor interaction^b^ | 6.64 × 10^-7^ |
| 25 | Insulin signaling pathway | 8.33 × 10^-7^ |
| 26 | ErbB signaling pathway | 9.33 × 10^-7^ |
| 27 | Gap junction | 1.10 × 10^-6^ |
| 28 | Amyotrophic lateral sclerosis (ALS) | 1.66 × 10^-6^ |
| 29 | GnRH signaling pathway | 2.02 × 10^-6^ |
| 30 | Calcium signaling pathway^b^ | 4.00 × 10^-6^ |
| 31 | Adipocytokine signaling pathway | 4.85 × 10^-6^ |
| 32 | Endocytosis | 5.20 × 10^-6^ |
| 33 | p53 signaling pathway | 5.28 × 10^-6^ |
| 34 | Long-term potentiation | 5.35 × 10^-6^ |
| 35 | Chemokine signaling pathway | 5.35 × 10^-6^ |
| 36 | Phosphatidylinositol signaling system | 7.59 × 10^-6^ |
| 37 | VEGF signaling pathway | 7.59 × 10^-6^ |
| 38 | Natural killer cell mediated cytotoxicity | 9.05 × 10^-6^ |
| 39 | Progesterone-mediated oocyte maturation | 1.33 × 10^-5^ |
| 40 | TGF-beta signaling pathway | 1.36 × 10^-5^ |
| 41 | Jak-STAT signaling pathway | 1.69 × 10^-5^ |
| 42 | T cell receptor signaling pathway | 3.70 × 10^-5^ |
| 43 | Leukocyte transendothelial migration | 5.53 × 10^-5^ |
| 44 | Tight junction | 9.86 × 10^-5^ |

^a^Adjusted *P*-value was calculated by hypergeometric test following by Benjamini-Hochberg multiple testing correction; ^b^Pathway was enriched in 72 genes.
