# Supplementary material for: Uncovering MicroRNA and Transcription Factor Mediated Regulatory Networks in Glioblastoma
Source: PLoS Comput Biol. 2012 Jul 19;8(7):e1002488. doi: 10.1371/journal.pcbi.1002488 (PMC3400583; doi:10.1371/journal.pcbi.1002488)
Supplement: Table S6 — Significantly enriched KEGG pathways in the 176 genes. (DOC) [file pcbi.1002488.s016.doc]

**Table S6.** Significantly enriched KEGG pathways in the 176 genes.

| **Rank** | **Pathway** | **Adjusted**  ***P*-value^a^** | **Rank** | **Pathway** | **Adjusted**  ***P*-value** |
| --- | --- | --- | --- | --- | --- |
| 1 | Pathways in cancer^b^ | 2.03 × 10^-24^ | 33 | Dorso-ventral axis formation | 5.20 × 10^-6^ |
| 2 | Focal adhesion^b,c^ | 6.01 × 10^-18^ | 34 | GnRH signaling pathway | 5.69 × 10^-6^ |
| 3 | Prostate cancer^b^ | 3.23 × 10^-14^ | 35 | Adipocytokine signaling pathway | 1.23 × 10^-5^ |
| 4 | Melanoma^b^ | 7.59 × 10^-14^ | 36 | Calcium signaling pathway | 1.23 × 10^-5^ |
| 5 | Neurotrophin signaling pathway | 1.17 × 10^-12^ | 37 | p53 signaling pathway | 1.36 × 10^-5^ |
| 6 | Glioma^b^ | 1.17 × 10^-12^ | 38 | Long-term potentiation | 1.39 × 10^-5^ |
| 7 | MAPK signaling pathway | 2.16 × 10^-12^ | 39 | Long-term depression | 1.39 × 10^-5^ |
| 8 | Colorectal cancer^b^ | 1.06 × 10^-11^ | 40 | Endocytosis^b^ | 1.53 × 10^-5^ |
| 9 | ECM-receptor interaction^b,c^ | 2.80 × 10^-10^ | 41 | VEGF signaling pathway | 1.93 × 10^-5^ |
| 10 | Non-small cell lung cancer | 2.80 × 10^-10^ | 42 | Phosphatidylinositol signaling system | 1.93 × 10^-5^ |
| 11 | Small cell lung cancer | 2.80 × 10^-10^ | 43 | Tight junction | 2.29 × 10^-5^ |
| 12 | Regulation of actin cytoskeleton^c^ | 3.50 × 10^-10^ | 44 | TGF-beta signaling pathway | 3.57 × 10^-5^ |
| 13 | Renal cell carcinoma^b^ | 1.72 × 10^-9^ | 45 | Jak-STAT signaling pathway | 4.99 × 10^-5^ |
| 14 | Pancreatic cancer | 2.01 × 10^-9^ | 46 | Melanogenesis | 7.35 × 10^-5^ |
| 15 | Chronic myeloid leukemia | 2.61 × 10^-9^ | 47 | T cell receptor signaling pathway | 9.44 × 10^-5^ |
| 16 | Endometrial cancer | 5.61 × 10^-9^ | 48 | Epithelial cell signaling in Helicobacter pylori infection | 0.0001 |
| 17 | Adherens junction | 8.74 × 10^-8^ | 49 | Thyroid cancer | 0.0003 |
| 18 | Fc epsilon RI signaling pathway | 9.90 × 10^-8^ | 50 | B cell receptor signaling pathway | 0.0003 |
| 19 | Type II diabetes mellitus | 1.03 × 10^-7^ | 51 | Apoptosis | 0.0005 |
| 20 | Axon guidance | 1.48 × 10^-7^ | 52 | Fc gamma R-mediated phagocytosis | 0.0008 |
| 21 | Gap junction | 2.11× 10^-7^ | 53 | Notch signaling pathway | 0.001 |
| 22 | Acute myeloid leukemia | 3.96 × 10^-7^ | 54 | mTOR signaling pathway | 0.0013 |
| 23 | Leukocyte transendothelial migration^c^ | 1.24 × 10^-6^ | 55 | Hedgehog signaling pathway | 0.0017 |
| 24 | Bladder cancer | 1.73 × 10^-6^ | 56 | RIG-I-like receptor signaling pathway | 0.0032 |
| 25 | Cell cycle | 1.98 × 10^-6^ | 57 | Wnt signaling pathway | 0.0034 |
| 26 | Chemokine signaling pathway | 2.22 × 10^-6^ | 58 | Toll-like receptor signaling pathway | 0.0083 |
| 27 | ErbB signaling pathway | 2.60 × 10^-6^ | 59 | Prion diseases | 0.0092 |
| 28 | Cytokine-cytokine receptor interaction^b^ | 2.60 × 10^-6^ | 60 | Lysosome^c^ | 0.0119 |
| 29 | Progesterone-mediated oocyte maturation | 2.60 × 10^-6^ | 61 | Inositol phosphate metabolism | 0.0203 |
| 30 | Insulin signaling pathway | 2.60 × 10^-6^ | 62 | Basal cell carcinoma | 0.0207 |
| 31 | Natural killer cell mediated cytotoxicity | 2.60 × 10^-6^ | 63 | NOD-like receptor signaling pathway | 0.0255 |
| 32 | Amyotrophic lateral sclerosis (ALS) | 4.23 × 10^-6^ | 64 | Complement and coagulation cascades | 0.0306 |

^a^Adjusted *P*-value was calculated by a hypergeometric test followed by Benjamini-Hochberg multiple testing correction.

^b^Pathway was enriched in the 32 common genes existing in both 3-node FFLs and 4-node FFLs.

^c^Pathway was enriched in the 23 unique genes existing in 4-node FFLs.
