# Supplementary material for: Uncovering MicroRNA and Transcription Factor Mediated Regulatory Networks in Glioblastoma
Source: PLoS Comput Biol. 2012 Jul 19;8(7):e1002488. doi: 10.1371/journal.pcbi.1002488 (PMC3400583; doi:10.1371/journal.pcbi.1002488)
Supplement: Table S8 — miRNAs potentially involved in GBM-specific Notch signaling pathway. (DOC) [file pcbi.1002488.s018.doc]

**Table S8. miRNAs potentially involved in GBM-specific Notch signaling pathway .**

| **miRNA** | **Degree in Notch-specific subnetwork** |
| --- | --- |
| hsa-miR-9 | 17 |
| hsa-miR-92b | 16 |
| hsa-miR-137 | 12 |
| hsa-miR-219-5p | 12 |
| hsa-miR-107 | 11 |
| hsa-miR-25 | 8 |
| hsa-miR-124 | 7 |
| hsa-miR-195 | 6 |
| hsa-miR-30e | 6 |
| hsa-miR-34a | 6 |
| hsa-miR-495 | 6 |
| hsa-miR-128 | 5 |
| hsa-miR-26b | 5 |
| hsa-miR-144 | 4 |
| hsa-miR-340 | 4 |
| hsa-miR-375 | 4 |
| hsa-miR-129-5p | 3 |
| hsa-miR-133a | 3 |
| hsa-miR-133b | 3 |
| hsa-miR-143 | 3 |
| hsa-miR-15b | 3 |
| hsa-miR-16 | 3 |
| hsa-miR-29c | 3 |
| hsa-miR-30d | 3 |
| hsa-miR-103 | 2 |
| hsa-miR-106b | 2 |
| hsa-miR-24 | 2 |
| hsa-miR-29b | 2 |
| hsa-miR-30b | 2 |
| hsa-miR-30c | 2 |
| hsa-miR-34c-5p | 2 |
| hsa-miR-93 | 2 |
