# Supplementary material for: Uncovering MicroRNA and Transcription Factor Mediated Regulatory Networks in Glioblastoma
Source: PLoS Comput Biol. 2012 Jul 19;8(7):e1002488. doi: 10.1371/journal.pcbi.1002488 (PMC3400583; doi:10.1371/journal.pcbi.1002488)
Supplement: Text S1 — Compiling glioblastoma-related genes (GBM-related genes) from multiple datasets. (DOC) [file pcbi.1002488.s019.doc]

Text S1. Compiling glioblastoma-related genes (GBM-related genes) from multiple datasets

COSMIC (Catalogue of Somatic Mutations in Cancer) is currently the most comprehensive global resource accessing world literature on somatic mutations in human cancer [1]. We downloaded the COSMIC gene data (version 51, February 7, 2011) from ftp://ftp.sanger.ac.uk/pub/CGP/cosmic/data_export/genes/. For each gene, we first retained the samples that are diagnosed as GBM and then tabulated the number of these samples (*N*) and the number of samples with positive mutation records (*n*). We required genes that have a number of samples with positive mutations of no less than 2 and a percentage (*n*/*N*) no less than 3%. Thus, we obtained 179 genes.

Recently, the National Cancer Institute (NCI) and the National Human Genome Research Institute (NHGRI) have launched the Cancer Genome Atlas (TCGA) project to systematically explore the entire spectrum of genomic changes involved in human cancer using genome analysis technologies, including large-scale genome sequencing. Through the sequencing of 91 matched tumor–normal pairs, 223 genes with 453 validated non-silent somatic mutations were discovered. We retrieved them from supplementary Table 6 of the TCGA [2008] [2].

Parson et al. [3] integrated the mutational data from sequencing and analyses of copy number alterations and identified 42 genes that accumulated mutations at significant frequencies. We retrieved them from supplementary Table 7 of the paper.

In addition to above resources, there are other resources that include gene mutations relative to GBM, such as OMIM and GAD. OMIM (Online Mendelian Inheritance in Man) is a database of human genes and genetic disorders [4]. We obtained 3 GBM-related genes with 3 evidences from OMIM. GAD (The Genetic Association database) database is an archive of summary data of published human genetic association studies of numerous common disease types, which contains curated information on candidate gene studies and, more recently, on genome wide association studies [5]. We obtained 6 GBM-related genes with positive association reports from the GAD.

There are 2 glioma genome-wide association (GWA) studies have been reported to date. Shete et al. [6] conducted a meta-analysis of two GWA studies that involved the genotyping of 454,576 tagging SNPs in a total of 1,878 glioma cases and 3,670 controls, with validation in 3 additional independent series totaling 2,545 cases and 2,953 controls. The second independent GWA study of glioma reported was based on an analysis of 275,895 SNPs in 692 adult patients with high-grade glioma and 3,992 controls with a replication series of 176 high-grade glioma cases and 174 controls [7]. We manually extracted 6 risk genes (*TERT*, *CCDC26*, *CDKN2A*, *CDKN2B*, *RTEL1*, and *PHLDB1*) for GBM.

Supplementary References

1. Forbes SA, Bindal N, Bamford S, Cole C, Kok CY, et al. (2011) COSMIC: mining complete cancer genomes in the Catalogue of Somatic Mutations in Cancer. Nucleic Acids Res 39: D945-950.

2. TCGA (2008) Comprehensive genomic characterization defines human glioblastoma genes and core pathways. Nature 455: 1061-1068.

3. Parsons DW, Jones S, Zhang X, Lin JC, Leary RJ, et al. (2008) An integrated genomic analysis of human glioblastoma multiforme. Science 321: 1807-1812.

4. Amberger J, Bocchini CA, Scott AF, Hamosh A (2009) McKusick's Online Mendelian Inheritance in Man (OMIM). Nucleic Acids Res 37: D793-796.

5. Becker KG, Barnes KC, Bright TJ, Wang SA (2004) The genetic association database. Nat Genet 36: 431-432.

6. Shete S, Hosking FJ, Robertson LB, Dobbins SE, Sanson M, et al. (2009) Genome-wide association study identifies five susceptibility loci for glioma. Nat Genet 41: 899-904.

7. Wrensch M, Jenkins RB, Chang JS, Yeh RF, Xiao Y, et al. (2009) Variants in the CDKN2B and RTEL1 regions are associated with high-grade glioma susceptibility. Nat Genet 41: 905-908.
