# Supplementary material for: Uncovering MicroRNA and Transcription Factor Mediated Regulatory Networks in Glioblastoma
Source: PLoS Comput Biol. 2012 Jul 19;8(7):e1002488. doi: 10.1371/journal.pcbi.1002488 (PMC3400583; doi:10.1371/journal.pcbi.1002488)
Supplement: Text S2 — Pfam annotation used to test whether the target protein in each merged FFL tend to belong to same protein family. (DOC) [file pcbi.1002488.s020.doc]

**Text S2. Pfam annotation**

To test whether the target proteins in each merged FFL tend to belong to same protein family more often than randomly selected proteins, we explored the protein family annotation from the Pfam database [1]. We first annotated all the 153 genes from 3-node FFLs with the Pfam database. Among the 153 genes, 148 genes have been annotated by Pfam. For each merged FFL that had at least n (n>1) targets and formed (n ×(n-1))/2 target pairs, we counted the number of pairs that had at least one of the same protein family annotations; these FFLs did not. For instance, for the TF-FFLs in 3-node FFLs, 166 target pairs in total shared at least one Pfam annotation while 3,286 target pairs did not share any Pfam annotations. To obtain a random background of Pfam sharing distribution from any two proteins, we randomly sampled 148 genes from 17,990 coding genes (from Entrez Gene database) with Pfam annotations; we repeated this step 1,000 times. From the random background for any protein pairs, 18,736 protein pairs shared Pfam annotation and 2,156,864 proteins pairs did not share Pfam annotation. For 4-node FFLs, we adopted a similar method to perform the process. Then, we performed Fisher's exact test to calculate *P*-values.

# Supplementary References

1. Finn RD, Tate J, Mistry J, Coggill PC, Sammut SJ, et al. (2008) The Pfam protein families database. Nucleic Acids Res 36: D281-288.
